# Identification of activated enhancers and linked transcription factors in breast, prostate, and kidney tumors by tracing enhancer networks using epigenetic traits

**DOI:** 10.1186/s13072-016-0102-4

**Published:** 2016-11-09

**Authors:** Suhn Kyong Rhie, Yu Guo, Yu Gyoung Tak, Lijing Yao, Hui Shen, Gerhard A. Coetzee, Peter W. Laird, Peggy J. Farnham

**Affiliations:** 1 Department of Biochemistry and Molecular Medicine and the Norris Comprehensive Cancer Center, Keck School of Medicine, University of Southern California, 1450 Biggy Street, NRT G511B, Los Angeles, CA 90089-9601 USA; 2 Van Andel Research Institute, Grand Rapids, MI 49503 USA

**Keywords:** DNA methylation, Epigenetics, Enhancer, Transcription factor, Networks

## Abstract

**Background:**

Although technological advances now allow increased tumor profiling, a detailed understanding of the mechanisms leading to the development of different cancers remains elusive. Our approach toward understanding the molecular events that lead to cancer is to characterize changes in transcriptional regulatory networks between normal and tumor tissue. Because enhancer activity is thought to be critical in regulating cell fate decisions, we have focused our studies on distal regulatory elements and transcription factors that bind to these elements.

**Results:**

Using DNA methylation data, we identified more than 25,000 enhancers that are differentially activated in breast, prostate, and kidney tumor tissues, as compared to normal tissues. We then developed an analytical approach called Tracing Enhancer Networks using Epigenetic Traits that correlates DNA methylation levels at enhancers with gene expression to identify more than 800,000 genome-wide links from enhancers to genes and from genes to enhancers. We found more than 1200 transcription factors to be involved in these tumor-specific enhancer networks. We further characterized several transcription factors linked to a large number of enhancers in each tumor type, including GATA3 in non-basal breast tumors, HOXC6 and DLX1 in prostate tumors, and ZNF395 in kidney tumors. We showed that HOXC6 and DLX1 are associated with different clusters of prostate tumor-specific enhancers and confer distinct transcriptomic changes upon knockdown in C42B prostate cancer cells. We also discovered de novo motifs enriched in enhancers linked to ZNF395 in kidney tumors.

**Conclusions:**

Our studies characterized tumor-specific enhancers and revealed key transcription factors involved in enhancer networks for specific tumor types and subgroups. Our findings, which include a large set of identified enhancers and transcription factors linked to those enhancers in breast, prostate, and kidney cancers, will facilitate understanding of enhancer networks and mechanisms leading to the development of these cancers.

**Electronic supplementary material:**

The online version of this article (doi:10.1186/s13072-016-0102-4) contains supplementary material, which is available to authorized users.

## Background

A single genome can give rise to several hundred distinct cell types that are genetically identical but display different epigenetic marks at regulatory elements, leading to altered gene expression. There are two main types of regulatory elements involved in transcriptional activation, promoters and enhancers. Promoters are defined as a relatively small region surrounding a transcription start site (TSS) of a gene and are critical for basal transcription of that gene. Enhancers are regulatory elements, containing multiple transcription factor (TF) binding sites, which can be far upstream or downstream of the gene they regulate [1]. Of note, the state most consistently linked to cellular identity is the ‘active enhancer’ state [2, 3]. In addition, previous studies have shown that epigenetic changes at enhancers are significantly better than those at promoters for predicting expression changes of target genes in cancer [4, 5].

Recent studies from the Encyclopedia of DNA Elements (ENCODE) and the Roadmap Epigenome Mapping Consortium (REMC) have shown that more than ten thousand enhancers can be identified using epigenomic marks in a given cell line or tissue [6, 7]. However, it is not clear whether all of these enhancers are functional [8] or which gene is regulated by each enhancer. One enhancer may regulate multiple genes, one gene may be regulated by multiple enhancers, and an enhancer does not always regulate the nearest gene. In addition, we do not have a complete understanding as to which TFs bind to and activate each enhancer in a particular cell type. Therefore, it is difficult to a priori develop a detailed transcriptional regulatory network for a given cell type [1, 9].

In this study, we have used known enhancer regions and have also performed Chromatin Immunoprecipitation (ChIP) and Formaldehyde-Assisted Isolation of Regulatory Elements (FAIRE) assays to annotate additional cell type-specific enhancers. Using these enhancer regions, along with DNA methylation data generated as part of The Cancer Genome Atlas (TCGA), we have identified enhancers that are activated or inactivated in breast, prostate, and kidney tumor tissues. To facilitate understanding of enhancer networks deregulated in tumors, we have developed an approach called Tracing Enhancer Networks using Epigenetic Traits (TENET), which identifies enhancer and gene expression relationships (links) genome-wide. Using TENET, with epigenomic and RNA expression data from breast, prostate, and kidney tumor and normal tissue samples, we found more than 25,000 differentially activated enhancers and more than 1200 transcription factors involved in tumor-specific enhancer networks. For example, we found that hundreds of tumor-specific enhancers are linked to *GATA3* overexpression in non-basal breast tumors. We showed that *HOXC6* and *DLX1*, independent prognostic markers of prostate tumors [10], are associated with distinct clusters of tumor-specific enhancers and transcriptomic changes in C42B prostate cancer cells upon knockdown of *HOXC6* and *DLX1*. We also discovered de novo motifs specifically enriched in enhancers linked to *ZNF395* in kidney tumors. Our findings, which include a large set of identified enhancers and TFs linked to those enhancers in breast, prostate, and kidney cancers, will facilitate understanding of disordered epigenetic regulation and enhancer networks in tumor types and subgroups.

## Results

### Identification of differentially methylated enhancers in breast, prostate, and kidney tumor tissues

Technologies such as ChIP, FAIRE, and DNaseI assays combined with sequencing [11] are generally used to identify enhancers in cell lines. However, these assays are not amenable for use with tissue samples because they require a large number of cells, are time consuming to perform, and do not work well with frozen tissues. However, the analysis of DNA methylation using arrays is easier, works well with frozen tissues, and can be performed using very few cells [12]. If an enhancer region is unmethylated, it corresponds to open chromatin that can be bound by TFs and is given an active enhancer status. On the other hand, if an enhancer region is methylated, it reflects closed chromatin that is not bound by TFs and is given an inactive enhancer state.

**Fig. 1:**
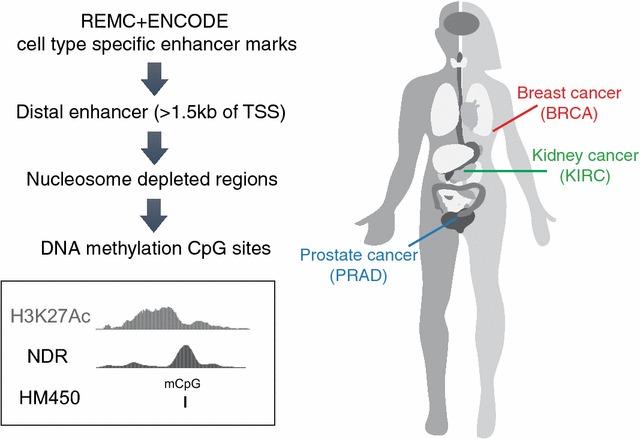
Study design. To define genomic regions for analysis of enhancer activity in tumor samples, we used the genomic coordinates of enhancers identified by REMC and ENCODE for 98 tissues or cell lines, plus genomic coordinates of additional H3K27Ac ChIP-seq peaks from several cancer cell lines and normal cells for breast, prostate, and kidney. We then selected the subset of these regulatory elements that are located >1.5 kb from a known transcription state site (TSS), as defined using GENCODE v19. We further narrowed the regions by intersecting with the set of ENCODE Master DNaseI-seq peaks from 125 tissues or cell lines or DNaseI-seq, FAIRE-seq, or NOMe-seq peaks of corresponding cell types (Additional file 2: Table S1). The HM450 array probes that overlapped the narrowed enhancer regions were then used to study enhancer activity in normal and tumor tissues

To identify activated and inactivated enhancers specific to breast, prostate, and kidney tumor tissue samples, we assembled a large set of genomic coordinates that includes regions previously identified as distal regulatory elements by ENCODE and REMC [6, 7] as well as enhancer locations derived from H3K27Ac ChIP-seq data specifically generated in our laboratory for this study (e.g., H3K27Ac ChIP-seq for MCF7, MDAMB231, and MCF10A breast cells and for C42B and RWPE1 prostate cells). Because recent studies have shown that a nucleosome-depleted region (NDR) flanked on each side by a nucleosome having the active enhancer histone mark H3K27Ac is where TFs actually bind [5, 13], we used public and newly generated Nucleosome Occupancy and Methylome Sequencing (NOMe-seq), DNaseI-seq, and FAIRE-seq datasets to further narrow enhancer regions (see Additional file 1: Supplementary Methods for a detailed description of the creation of the enhancer file and Additional file 2: Table S1 for a list of datasets). These narrowed regions represent the functional (TF binding) compartment of the larger regions defined by ChIP-seq data. The subset of these narrowed TF binding regulatory regions represented by probes on the Illumina HM450 array was then identified for use in our study (Fig. 1).

The DNA methylation profiles of the probes representing the narrowed enhancer regions in tumor and normal tissue samples were compared using 641 tumors and 66 normals for breast invasive carcinoma (BRCA), 333 tumors and 19 normals for prostate adenocarcinoma (PRAD), and 318 tumors and 24 normals for kidney renal clear cell carcinoma (KIRC) from TCGA. A major problem when characterizing tissues is the purity of each sample. For instance, TCGA has shown that the proportion of normal cells and immune cells that are intermixed with cancerous cells in a tumor tissue sample can greatly affect the results of genetic and epigenetic analyses. Specifically, DNA methylation analysis of prostate tumors was shown to be heavily confounded by tumor purity, with leukocyte infiltration being a major factor of tissue contamination [14]. To alleviate the purity effects in our analyses, we assessed the DNA methylation levels at enhancer regions in normal cells from the same cell type as the tumors, as well as in other normal cells such as leukocytes, smooth muscles, and fibroblasts. For this study, we only classified probes as hypermethylated in tumors compared to normal if these same probes did not also have high DNA methylation levels in leukocytes; similarly, we only classified probes as hypomethylated in tumors compared to normal if they did not also have low levels of DNA methylation in leukocytes (Additional file 1: Figures S1, S2). Although this winnowing likely removed some probes that displayed tumor-specific methylation changes, we felt that it was best to reduce potential false positives from our analyses.

**Fig. 2:**
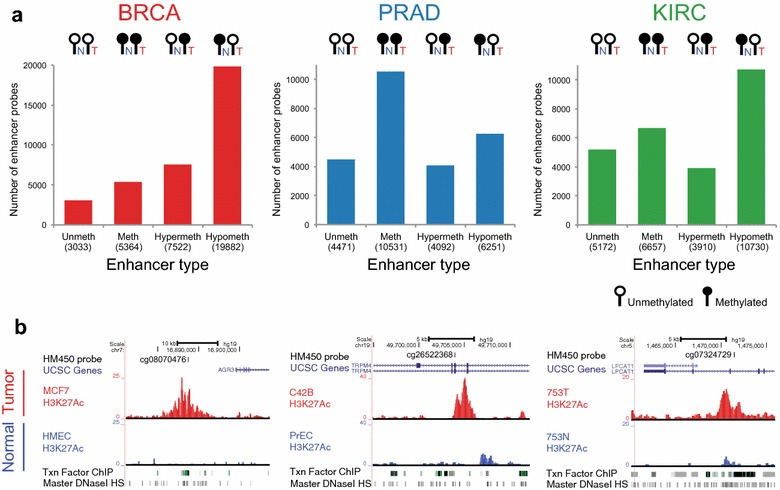
Identification of differentially methylated enhancer regions. **a** Differentially methylated enhancer probes located in epigenetically defined enhancers were identified by using DNA methylation profiles from TCGA for breast (BRCA), prostate (PRAD), and kidney (KIRC) tumor tissues. Unmeth: enhancer probes unmethylated in both normal and tumor samples; Meth: enhancer probes methylated in both normal and tumor samples; Hypermeth: enhancer probes unmethylated in normals, but methylated in tumors; Hypometh: enhancer probes methylated in normals, but unmethylated in tumors; the number of enhancer probes for each category is shown in parentheses. **b** Examples of hypomethylated enhancers (i.e., tumor-specific enhancers) are shown for BRCA (*center*), PRAD (*left*), and KIRC (*right*). Genome browser screen shots show genomic coordinates, HM450 probe location, UCSC genes, H3K27Ac ChIP-seq tracks in tumor (MCF7, C42B, and 753T) and normal (HMEC, PrEC, and 753N) cells, the ENCODE layered ChIP-seq track for 161 TFs, and the ENCODE Master DNaseI hypersensitive site track for 125 cell types

 Probes were categorized to four enhancer groups: unmethylated in both normal and tumor samples (this group is termed “always unmethylated” and represents enhancers active in both normal and tumor samples), methylated in both normal and tumor samples (this group is termed “always methylated” and represents enhancers inactive in both normal and tumor samples), hypermethylated in tumors as compared to normal samples (this group is termed “normal-specific enhancers” and represents enhancers active in normal but inactive in tumors), and hypomethylated in tumors as compared to normal tissues (this group is termed “tumor-specific enhancers” and represents enhancers inactive in normal and active in tumors) (Fig. 2a). We identified more than 50,000 probes that are differentially methylated, representing ~25,000 different enhancers that are gained or lost in the BRCA, PRAD, or KIRC samples (Additional file 3: Table S2). Interestingly, different fractions of probes belonged to each enhancer group across tumor types. For example, we identified relatively more “always methylated” probes in PRAD than in BRCA and relatively more hypomethylated probes in BRCA and KIRC than in PRAD. When we further compared the activity state of enhancers in the normals versus tumors for each tumor type, we found both common and tumor type-specific normal-to-tumor activity changes at these enhancers. For example, among the ~6000–20,000 hypomethylated enhancer probes from the three tumor types (corresponding to enhancers gained in tumors), only 2514 probes identified tumor-specific enhancers in all 3 tumor types, suggesting that there are critical TFs in each tissue type that drive distinct breast, prostate, and kidney tumor development (Additional file 1: Figure S3). Examples of tumor-specific enhancers identified using the TCGA DNA methylation data that were confirmed to have tumor-specific H3K27Ac ChIP signals in appropriate tumor cell lines are shown in Fig. 2b.

### Identification of linked genes for differentially methylated enhancers

To understand how enhancer activity may contribute to cancer initiation or progression, we associated genome-wide gene expression changes with gain or loss of enhancer activity, using an approach called TENET (Additional file 1: Figures S1, S2, S4). Enhancers are generally considered to regulate expression of their direct target genes in a positive direction. Therefore, possible direct targets are those in which a gained activity state of the enhancer is associated with an increase in gene expression. Of course, there will be many other genes whose expression is also positively associated with the activity of an enhancer (e.g., genes whose expression increases as a consequence of the increased expression of the direct target gene). The set of genes (direct and indirect targets) whose expression is positively associated with the normal-specific activity of an enhancer is indicated as E^N^:G^+^, and the set of genes whose expression is positively associated with the tumor-specific activity of an enhancer is indicated as E^T^:G^+^ (Additional file 1: Figure S4 top). Conversely, genes whose expression is negatively correlated with enhancer activity are not likely to be direct targets but instead may show decreased expression due to changed expression of, for example, a transcriptional repressor that is a direct target. The set of genes whose expression is negatively associated with the normal-specific activity of an enhancer is indicated as E^N^:G^**−**^, and the set of genes whose expression is negatively associated with the tumor-specific activity of an enhancer is indicated as E^T^:G^**−**^ (Additional file 1: Figure S4 bottom). In total, we identified ~800,000 enhancer:gene links in these 4 categories (E^N^:G^+^, E^T^:G^+^, E^N^:G^**−**^, and E^T^:G^−^); see Additional file 4: Table S3.

Of high interest in our study of regulatory regions involved in tumor development is the E^T^:G^+^ subset of tumor-specific enhancers that are positively linked to gene expression. Among the tumor-specific enhancer probes (19,882 for BRCA, 6251 for PRAD and 10,730 for KIRC; see Fig. 2a), only 10–20% were positively linked (directly or indirectly) to genes. We identified 127,725 E^T^:G^+^ links between 4334 probes and 6948 genes for BRCA, 25,428 E^T^:G^+^ links between 1120 probes and 5017 genes for PRAD, and 117,557 E^T^:G^+^ links between 2535 probes and 6629 genes for KIRC (Additional file 4: Table S3, Additional file 1: Figure S5).

**Fig. 3:**
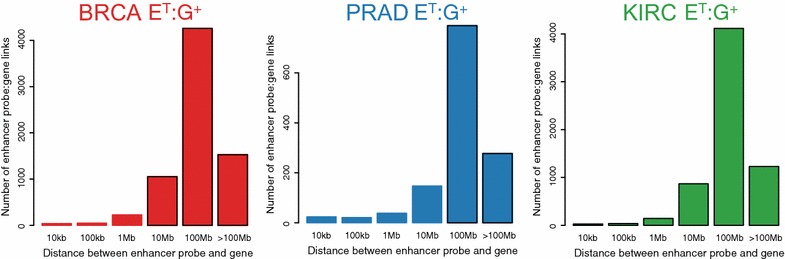
Distribution of enhancer probe:gene links on the same chromosome. Shown is the number of enhancer probe to gene links (E^T^:G^+^) on the same chromosome by distance in BRCA (*left*, *red*), PRAD (*center*, *blue*), and KIRC (*right*, *green*)

As noted above, the links include not only direct target genes of the enhancers but also genes whose expression is indirectly regulated by an enhancer due to secondary or downstream effects. Hi-C and tethered chromatin capture suggest that direct interactions between enhancers and promoters mostly occur on the same chromosome within topologically associating domains, which are about 1 Mb in length and include 4–10 genes and several hundred enhancers [15]. To identify potential direct target genes of the enhancer probes, the distance between the enhancer probes and linked genes that are on the same chromosome was measured. For example, among the 127,725 E^T^:G^+^ links in BRCA, 7153 are within the same chromosome. Of these, 313 E^T^:G^+^ enhancer:gene links were found within a 1-Mb region. For PRAD and KIRC, 83 and 212 E^T^:G^+^ enhancer:gene links were found within a 1-Mb region, respectively (Fig. 3; Additional file 1: Figure S6, Additional file 5: Table S4).

### TFs associated with the activity of many tumor-specific enhancers

**Fig. 4:**
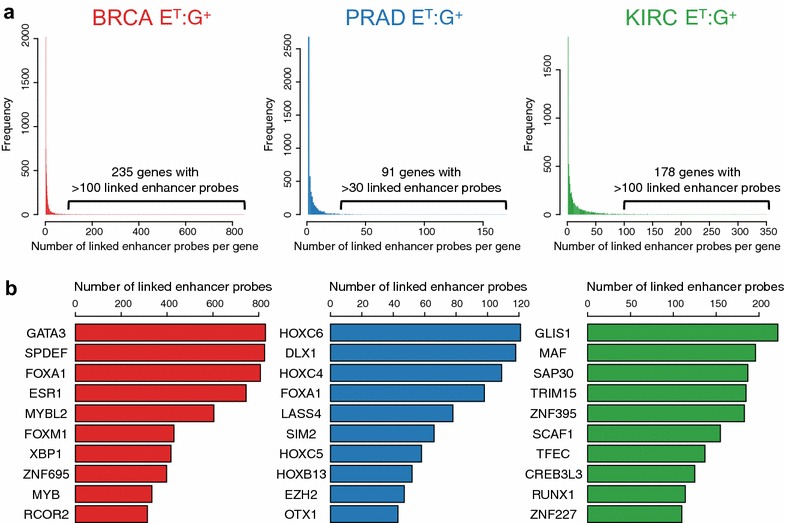
Identification of TFs associated with the activity of many enhancers. **a** Shown is the number of linked enhancer probes per gene in the E^T^:G^+^ category for BRCA (*left*), PRAD (*center*), and KIRC (*right*). **b** The top 10 TFs identified to be linked to a large number of enhancer probes for BRCA (*left*), PRAD (*center*), and KIRC (*right*)

Studies from the ENCODE project reported that the average number of enhancers directly interacting with a promoter via looping is 3.9 [9]. Although our analysis is not limited to direct interactions, the majority of genes are associated with fewer than 5 enhancer probes (Fig. 4a; Additional file 1: Figures S7, S8, Additional file 4: Table S3). However, strikingly, in each tumor type, a subset of genes is associated with many enhancer probes. For BRCA, among the 6948 genes whose expression is positively associated with activity of a tumor-specific enhancer probe, 235 genes were associated with more than 100 enhancer probes. For PRAD, among the 5017 whose expression is positively associated with activity of a tumor-specific enhancer probe, 91 genes were associated with more than 30 enhancer probes, and for KIRC, among the 6629 genes whose expression is positively associated with activity of a tumor-specific enhancer probe, 178 genes were associated with more than 100 enhancer probes. The links between genes and enhancers for those genes whose expression is positively associated with a large number of enhancer probes can be viewed in two ways. Either many enhancers regulate that gene, or perhaps more likely if the gene is a TF, then the association can be reversed. In other words, high expression of a TF can lead to increased occupancy (and hypomethylation) at target enhancers of the TF. For each tumor type, we identified a unique set of TFs that are linked to enhancers; for BRCA we identified 710 TFs, for PRAD we identified 540 TFs, and for KIRC we identified 731 TFs, for a union set of ~1200 TFs (Fig. 4b; Additional file 6: Table S5). Among those, for example, GATA3, SPDEF, FOXA1, and ESR1 are TFs linked to hundreds of enhancers in BRCA. Similarly, HOXC6, DLX1, and HOXC4 are top TFs linked to more than a hundred enhancers in PRAD, whereas GLIS1, MAF, SAP30, TRIM15, and ZNF395 are TFs linked to hundreds of enhancers in KIRC. We further investigated top TFs linked to hundreds of enhancers in breast, prostate, and kidney tumors.

### Characterization of TFs linked to breast tumor-specific enhancers

**Fig. 5:**
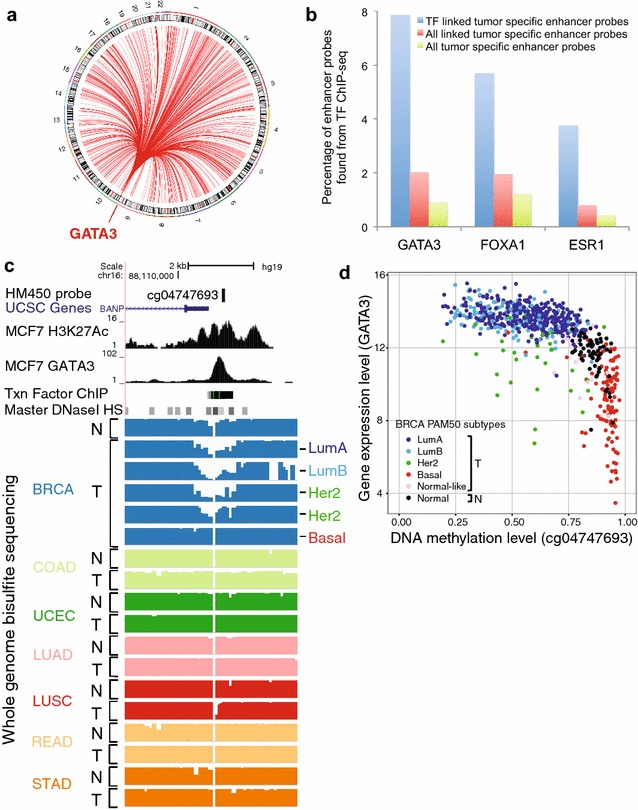
GATA3 is linked to many enhancers in breast tumor tissues. **a** Shown is a circos plot of the enhancers having an active state positively linked to expression of *GATA3*. **b** Percentage of all tumor-specific enhancers (*green*), all tumor-specific enhancers linked to genes (*red*), and tumor-specific enhancers linked to *GATA3*, *FOXA1*, or *ESR1* (*blue*) expression that overlap with TF ChIP-seq for GATA3, FOXA1, or ESR1. **c** Genome browser screen shots of an enhancer (containing probe cg04747693) having an active state in breast tumors, that is positively linked to expression of *GATA3*; the probe is located within a H3K27Ac and a GATA3 peak in MCF7 cells and in a hypomethylated region specifically found in non-basal breast cancer cells. **d** Shown is a scatterplot of the DNA methylation of the enhancer probe and *GATA3* expression in normal and different subtypes of breast tumor tissues

GATA3 is a well-studied TF that has a long history of association with breast cancer. Therefore, we investigated the enhancer to gene links identified above for GATA3 using publicly available ChIP-seq data from a breast cancer cell line (Additional file 2: Table S1). *GATA3* expression was associated with 829 breast tumor-specific enhancer probes located on many different chromosomes (Fig. 5a). We found that GATA3 ChIP-seq peaks from the MCF7 ER+ breast cancer cell line were statistically significantly enriched in the set of tumor-specific enhancers linked to *GATA3* in BRCA by TENET, as compared to all tumor-specific enhancers linked to genes or to all tumor-specific enhancers (Fisher exact test, adj. *p* value <4 × 10^−15^) (Fig. 5b); this pattern of enrichment of the appropriate ChIP-seq peaks in the TF-linked enhancer probe sets was also found for FOXA1 and ESR1. This analysis supports the conclusion that enhancers linked to a TF by TENET include a subset of enhancers that are bound by that TF. As an example, the *GATA3*-linked tumor-specific enhancer probe cg04747693 is within the H3K27Ac and GATA3 ChIP-seq signals in MCF7. When we investigated this region using whole genome bisulfite sequencing data from TCGA, we found that CpGs near the GATA3 peak were unmethylated in breast but not in the other tumor types. Importantly, this probe was specifically unmethylated in non-basal breast tumors (Fig. 5c), likely due to the higher expression of GATA3 in luminal, as compared to basal tumors (Fig. 5d). We recognize that the MCF7 ER+ breast cancer cell line does not well represent all of the heterogeneous 641 breast tumor tissue samples that we used, and thus the MCF7 ChIP-seq data cannot validate all of the tumor-specific enhancers we identified. Therefore, we performed motif enrichment analyses for the GATA3-linked enhancers and found that 24% of GATA3-linked enhancers contained GATA3 motifs, validating TENET predictions. In addition to the GATA3 motif, we also found that the GATA3-linked enhancers have known motifs of other transcription factors (e.g., FOXA, ESR1, TCF), which have been previously shown to work together with GATA3 in breast cancer [16, 17]. These results suggest that through TENET analyses, we can identify sets of TFs co-recruited to enhancers.

### Characterization of prostate tumor-specific enhancer networks

**Fig. 6:**
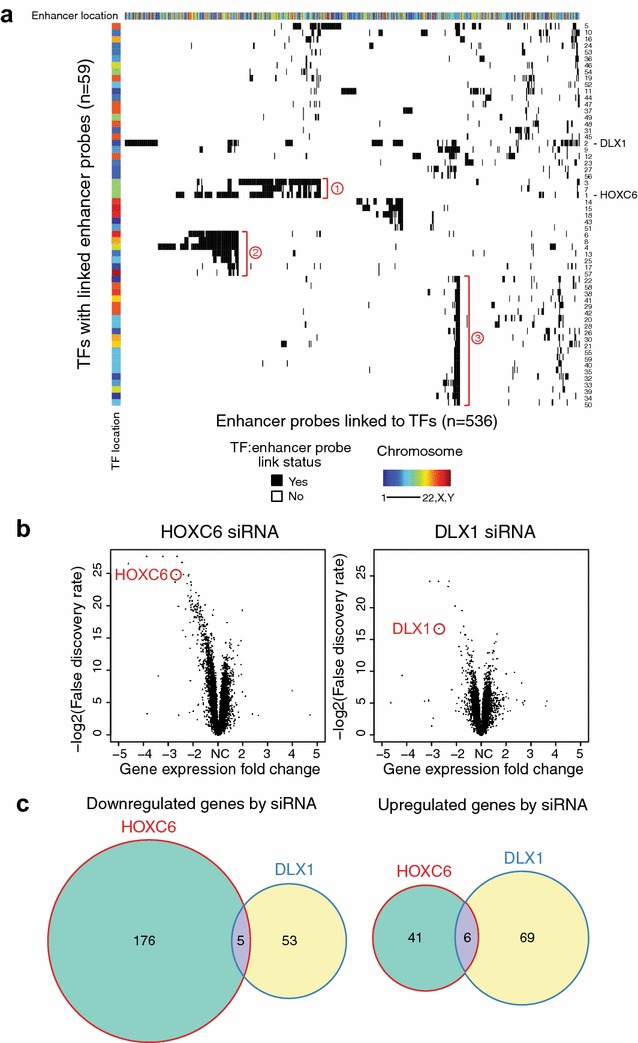
TFs linked to many tumor-specific enhancers in prostate tissues. **a** Unsupervised clustering of the enhancer probe:TF sets for PRAD for which a TF is associated with more than 10 hypomethylated (tumor-specific) enhancer probes. The rows indicate the 59 TFs, and the columns indicate the 536 hypomethylated enhancer probes linked to TFs; when there is a link, the cell is colored in *black*. On the top of the heatmap is shown the chromosomal location for each enhancer probe. On the *left side* of the heatmap is shown the chromosomal location for each TF. TF number on the *right side* indicates the TF rank, as determined by the number of linked enhancer probes for each TF (Additional file 6: Table S5). Three clusters of TFs that are linked to the same enhancers are marked by brackets with circled numbers. **b** Volcano plots identifying genes differentially expressed upon knockdown of HOXC6 and DLX1; triplicate control and knockdown samples were analyzed. **c** Venn diagrams of significantly down- or upregulated genes upon knockdown of HOXC6 and DLX1

Unlike the association of GATA3 with breast cancer, the TFs identified by TENET in prostate cancer have not been well studied. The top 2 TFs associated with the most tumor-specific enhancer probes in prostate tumors, HOXC6 and DLX1, are each associated with more than 100 tumor-specific enhancer probes (Fig. 4b). Interestingly, HOXC6 and DLX1 were recently identified as top markers for prostate cancer, but little is known about their target genes [10]. To further characterize the highly linked TFs in the E^T^:G^+^ category for PRAD, we asked whether they are associated with same or different enhancers. When we performed clustering of the enhancer probe:gene links for 59 different TFs linked to more than 10 enhancer probes, we found that many of the TFs shared subsets of linked enhancer probes. Several clusters of TFs that are associated with the same enhancers are marked by brackets with circled numbers in Fig. 6a. Cluster 1 contains HOXC6, HOXC4, and HOXC5, cluster 2 contains HOXB13 and FOXA1, and cluster 3 contains 19 TFs of which 8 are ZNFs (see Additional file 6: Table S5 for the numbered list of the 59 TFs). However, HOXC6 and DLX1 mostly do not share clusters of enhancer probes, suggesting that they regulate distinct sets of genes. To identify genes regulated by these TFs, we used siRNA (performed in triplicate) to reduce their expression in C42B prostate cancer cells, followed by RNA-seq (Fig. 6b, c; Additional file 7: Table S6, Additional file 8: Table S7). Interestingly, the sets of genes changed by knockdown of each TF are different, supporting the hypothesis that each TF has a distinct role in prostate cancer.

**Fig. 7:**
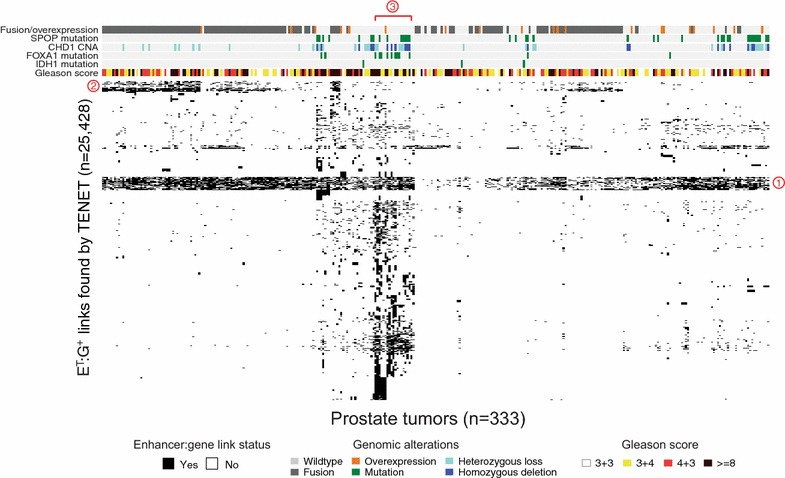
Heatmap of enhancer:gene links in prostate tumor tissues. Unsupervised clustering results using the E^T^:G^+^ links (*n* = 25,428) for prostate tumors (*n* = 333) with previously defined genomic alternations commonly found in prostate tumors and Gleason scores of the tumors [14]. Three clusters of E^T^:G^+^ links are marked by red-circled numbers

Because there is a substantial heterogeneity in prostate tumor samples, we felt it important to determine the enhancer to gene link states of each tumor sample to discover if any of the tumor-specific enhancers linked to genes are enriched in subsets of tumors having previously identified characteristics. For example, in PRAD, the most commonly found tumor subgroup has gene fusions involving members of the E26 transformation-specific (*ETS*) family of TFs, such as *ERG*, *ETV1*, *ETV4*, and *FLI1* [18]. Another common subgroup, which does not carry *ETS* fusion genes, may have either a mutation in the *SPOP gene* or a deletion of the *CHD1* gene [19]. Additionally, mutations of the *TP53*, *PTEN*, *FOXA1*, or *IDH1* genes occur in subgroups of prostate cancers [14]. The clinical behavior and progression of prostate cancers vary case by case [20], and an understanding of the mechanisms leading to the development of the different prostate cancer subgroups is in great demand. We therefore more closely examined the 25,428 E^T^:G^+^ links in different subsets of 333 prostate tumors (Fig. 7).

Interestingly, we detected a set of enhancer:gene links that are common across the tumors (e.g., the 1075 E^T^:G^+^ links in cluster 1). The 115 genes linked by these enhancers include genes that have been reported to be involved in prostate cancer development. One example of a gene which was associated with active states of enhancer probes across all prostate tumors is the *CAMKK2* gene, an AR-regulated gene that is an upstream activator of the AMP-dependent protein kinase (AMPK) and is involved in catabolic pathways and physiologically relevant processes such as cell cycle and cytoskeleton reorganization [21]. Gene ontology analyses of these 115 linked genes identified across all prostate tumors revealed that the category of sequence-specific DNA binding is enriched (Fisher exact test, adj. *p* value <3.1 × 10^−3^). Interestingly, *HOXC6* and *DLX1*, which we characterized above, were also found in these “common” links, suggesting that they may play a role in the development of the majority of prostate tumors. However, in addition to the “common” links, we identified more than 20,000 enhancer:gene links that are uniquely enriched in a particular subgroup of prostate tumors (Fig. 7). For example, cluster 2 of E^T^:G^+^ links is enriched in *ETS* fusion-positive tumors, whereas cluster 3 is enriched in tumors having a *FOXA1* mutation.

### ZNF395 linked enhancers in kidney cancers have common de novo motifs

**Fig. 8:**
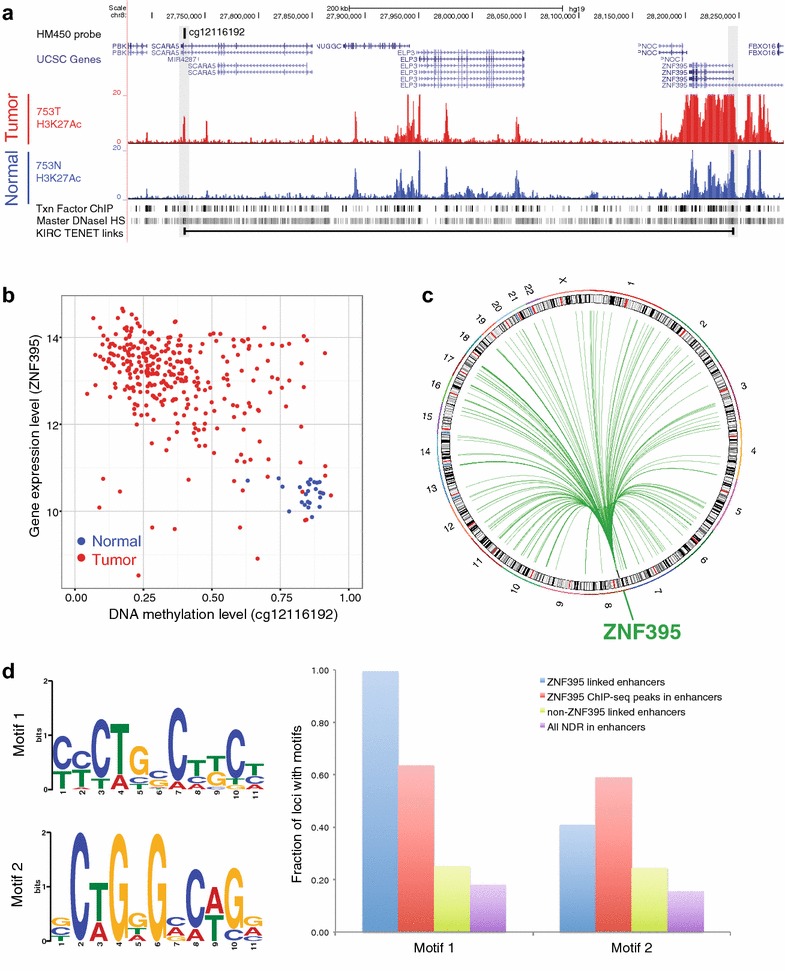
ZNF395-linked enhancers in kidney tumor tissues. **a** Genome browser screen shots near the tumor-specific enhancer probe cg12116192. From top, shown are the genomic coordinates, HM450 probe location, UCSC genes, H3K27Ac ChIP-seq tracks in tumor (753T) and normal (753N) cells, the ENCODE layered TF ChIP-seq track, the ENCODE Master DNaseI hypersensitive site track, and an intra-chromosomal TENET-identified link between the enhancer probe cg12116192 and the *ZNF395* gene; *left shaded* region is the enhancer probe cg12116192, and the *right shaded region* is the transcription start site of *ZNF395*. **b** Scatterplot of the DNA methylation level of the enhancer probe cg12116192 and *ZNF395* expression in normal and tumor kidney tissues. **c** Circos plot of the 183 enhancers having an active state positively linked to expression of the *ZNF395* gene in KIRC. **d** Logos of two de novo motifs identified in the 183 enhancers linked to *ZNF395* expression are shown on the *left*; fraction of regions with the two motifs in the 183 ZNF395-linked enhancers, in 7767 enhancers identified using a GFP antibody in K562 cells expressing a GFP-tagged ZNF395, in all linked enhancers identified in KIRC except those linked to ZNF395, and in all distal NDR regions used in this study

To follow up on our identification of TFs linked to hundreds of enhancers in kidney tumors, we further studied enhancers linked to ZNF395. *ZNF395*, which encodes a protein having one C2H2-type zinc finger domain, is overexpressed in various tumors including kidney cancer [22] and has been reported to be induced by hypoxia involved in IKK signaling [23]. The *ZNF395* gene is located at 8p21, and TENET identified hypomethylation of enhancer probe cg12116192 (located ~500 kb from the TSS of *ZNF395*) to be positively associated with the expression of the *ZNF395* gene (Fig. 8a, b), suggesting that hypomethylation of this probe may be responsible for the increased expression of *ZNF395* in kidney tumors. Although *ZNF395* has been repeatedly identified as a significant marker of renal cell carcinoma [24], very little functional characterization of this TF has been performed. As mentioned above, *ZNF395* expression level is positively associated with almost 200 hypomethylated enhancer probes that are located throughout the genome (Fig. 8c). ZNF395 has not been extensively studied, and there are no published ChIP-seq datasets or motifs for this TF. However, if the linked enhancers are in fact ZNF395-regulated enhancers, they may contain a common motif. Therefore, we performed a de novo motif search [25] on these 183 ZNF395-linked enhancers. Interestingly, we found that two motifs (*E*-value <3.2 × 10^−5^) are enriched; motif 1 is found in 182 of the 183 enhancers, and motif 2 is found in 75 of the 183 enhancers. However, less than 25% of enhancers that are linked to genes other than *ZNF395* in KIRC had these motifs and less than 20% of all NDRs distal from a TSS had the motifs (Fig. 8d). The fact that these motifs are found in essentially all of the ZNF395-linked enhancers suggests that they may be direct binding motifs for ZNF395. Although ZNF395 ChIP-seq data obtained using an antibody to the endogenous protein have not been published, ChIP-seq data obtained using a GFP antibody and K562 leukemia cells harboring a GFP-tagged ZNF395 are available as part of the ENCODE project. When we searched for motif 1 and motif 2 in the distal GFP-ZNF395 K562 ChIP-seq peak set, we found that 64% of the peaks contained motif 1 and 59% of the peaks contained motif 2. These results suggest that not only have we identified the DNA binding motif for ZNF395, they provide evidence that TENET can be used to identify enhancers and derive de novo motifs for TFs that have not yet been studied using ChIP-seq (Additional file 9: Table S8).

## Discussion

To understand the mechanisms underlying breast, prostate, and kidney cancers, we identified differentially active enhancers in breast, prostate, and kidney tumor tissues, as compared to normal tissues. By using an approach developed here called Tracing Enhancer Networks using Epigenetic Traits (TENET), which uses DNA methylation and gene expression levels to discover genome-wide links from enhancers to genes and from genes to enhancers, we discovered key TFs linked to tumor-specific enhancers (Fig. 4b, Additional file 6: Table S5). We further validated binding of the TFs GATA3, FOXA1 and ESR1 to the enhancers activated in breast cancers using publicly available ChIP-seq data. By performing knockdown assays and RNA-seq of the TFs HOXC6 and DLX1 in prostate cancer and annotating enhancer states in each prostate tumor sample, we found that expression of HOXC6 and DLX1 is highly linked to enhancers in the majority of prostate tumors, but they are associated with different enhancers and regulate distinct gene sets. We also revealed important TFs linked to enhancers activated in kidney cancer and further identified de novo motifs enriched in enhancers linked to ZNF395.

Previous studies have shown that all enhancers marked by active epigenomic marks may not regulate gene expression in the cells being studied [8, 26]. To prioritize enhancers which may possibly regulate gene expression among all enhancers marked by active epigenomic marks, we first identified ~50,000 probes (located within nucleosome-depleted subregions of enhancers) that are differentially methylated in breast, prostate, and kidney tumors, as compared to normal tissues; these probes correspond to ~25,000 different enhancer regions that have gained or lost activity in the tumor tissues. Because previous studies have shown a significant association between the DNA methylation level of an enhancer and the expression level of a direct target gene of the enhancer [27, 28], we then developed an approach (TENET) that identifies statistically significantly associated relationships (links) between DNA methylation and gene expression genome-wide using raw *p* values by calculating z scores, empirical *p* values, and Wilcoxon rank sum test *p* values. Although the number of enhancer to gene links can vary depending on the settings of cut-offs, in general, when we linked gene expression levels with enhancer activity, we found that only ~20% of the enhancer probes show a positive relationship between activity state and expression of a gene. Of these, ~40% of the enhancer probe:gene links are between enhancers and genes on the same chromosome, ~15% are within 10 MB of each other, and only ~5% of the links are between an enhancer and a gene located within 1 MB of each other. In total, we identified 608 enhancer probe:gene links (corresponding to 383 unique enhancers) in which the expression of a nearby gene (within 1 Mb) is positively associated with the activity of the enhancer (Additional file 5: Table S4); this set of links is the most likely to identify direct target genes. However, it is impossible to determine whether the associations are direct or indirect by comparing DNA methylation and gene expression changes. Chromosomal looping assays are often used to evaluate chromosomal interactions; however, the tissues analyzed here are not available for further experimental follow-up. Future studies will require the identification of tumor cell lines that show the appropriate enhancer activity:gene expression relationship (i.e., robust enhancer marks and high gene expression).

Although most genes and enhancers were involved in a relatively small number of links, we did identify some genes linked to hundreds of enhancers located throughout the genome. Although one may think that the most statistically significant enhancer to gene links would correspond to nearby, direct target genes, our results revealed that many links between an enhancer and a gene located far away or on another chromosome had very strong relationships. Many of these genes are TFs, some of which have previously been associated with the cancer in which the link was identified (Additional file 6: Table S5). For example, we identified GATA3 and FOXA1 as highly linked TFs in breast tumors. GATA3 and FOXA1 act as pioneer factors essential for mammary morphogenesis, and GATA3 is required for estradiol stimulation of cell cycle progression in breast cancer cells [29]. TENET identified HOXC6 and DLX1 as important TFs in PRAD. *HOXC6* is involved in epithelial cell proliferation, and loss of this gene induces apoptosis in prostate cancer cells [30, 31]. *DLX1* encodes a distal-less homeobox 1 protein that is reported to drive prostate cancer metastasis [32]. Upon independent knockdown of these genes in C42B prostate cancer cells, we found that for HOXC6, the top GO terms for downregulated genes were mitotic cell cycle and cell cycle (e.g., *CDKN2C*, *CDK16*, *IGFBP3*), and for DLX1, genes involved in proliferation and androgen-responsive genes were enriched in downregulated genes (e.g., *CDCA7*, *MAGOH*, *MAD2L1*). However, different genes were identified upon knockdown of *HOXC6* and *DLX1,* suggesting that each of these TFs has a distinct role in prostate cancer (Fig. 6c). This supports the recent finding that the combination of three genes (*HOXC6*, *DLX1*, and *TDRD1*) constitutes the top prognostic marker for prostate cancer [10]. Some of the TENET-identified TFs in KIRC (TFEC, RUNX1, ZNF395) have been previously linked to kidney cancer. For example, the dysregulation of *TFEC,* which belongs to the micropthalmia family of TFs, leads to renal cell carcinoma [33], *RUNX1* upregulation is an important factor for clear cell renal carcinoma survival [1, 34], and *ZNF395* is known to play a role in the pathogenesis of clear cell renal carcinoma [24], possibly affecting kidney cancer patient survival (Additional file 1: Figure S9). Our analyses using ChIP-seq data for TFs identified by TENET in BRCA and KIRC suggest that enhancer:gene links identified by this approach may help to identify specific TF binding sites and DNA binding motifs; this finding may be very beneficial for studies of TFs for which we do not have available antibodies for functional assays and for understanding TF-enhancer-gene networks (Additional file 1: Figure S10).

## Conclusions

In this study, we developed an approach (TENET) that alleviated tumor purity issues and then identified more than 800,000 enhancer probe to gene links in prostate, breast, and kidney tissue samples using only DNA methylation and RNA-seq data (Additional file 5: Table S4). We revealed TFs whose expression is linked to a large number of tumor-specific enhancers and further characterized selected TFs for each tumor type (e.g., BRCA: GATA3, FOXA1, ESR1, PRAD: HOXC6, DLX1, KIRC: ZNF395) and for specific tumor subgroups. However, there are limitations to our analyses. For example, currently there is no H3K27Ac ChIP-seq data and no whole genome bisulfite sequencing data available for the ~1400 tissue samples we used. Because the DNA methylation data for the normal and tumor tissues are from HM450 arrays, we can only investigate enhancers represented by these probes. For future studies, data from the Illumina EPIC array (which has more enhancer probes) or from whole genome bisulfite sequencing of normal and tumor tissues can be used with TENET to comprehensively identify tumor-specific changes in enhancer activity. We note that our approach can only identify enhancer to gene links that show changes in samples. Therefore, enhancers linked to genes that are expressed at a high level across normal and tumor samples (even if regulated by different enhancers in the tumors) as well as enhancers that are constitutively active across samples (even if they regulate different genes in the normals vs. tumors) cannot be identified. Finally, we stress that our approach can also be applied to studies beyond cancer to characterize enhancer networks for different types of case versus control datasets.

## Methods

### Cell culture growth conditions

The human prostate cancer C42B cells, obtained from ViroMed Laboratories (Minneapolis, MN, USA), were maintained in RPMI 1640 supplemented with 10% fetal bovine serum (FBS). The human immortalized normal prostate cell line RWPE1 (ATCC # CRL-11609) was grown according to the manufacturer’s recommendation. The human breast cancer MCF7 cells (ATCC # HTB-22) were grown in DMEM supplemented with 10% FBS. For estradiol stimulation, cells were grown in phenol red-free medium with charcoal stripped serum for several days and treated with 100 nM of estradiol for 45 min (as a control, ethanol was added instead of estradiol). The human immortalized normal breast cell line MCF10A (ATCC # CRL-10317) was maintained in DMEM/F12 with 5% horse serum, 100 units/ml penicillin, 0.1 mg/ml streptomycin, 0.5 ug/ml hydrocortisone, 100 ng/ml cholera toxin, 10 ug/ml insulin, and 20 ng/ml epidermal growth factor.

### ChIP-seq

In C42B, RWPE1, MCF7, and MCF10A cells, H3K27Ac ChIP assays were performed using H3K27Ac antibody (Cat # 39133 Lot # 21311004, Active Motif, Carlsbad, CA, USA or ab4729 Abcam, Cambridge, MA, USA), as previously described [5, 35]. Each ChIP-seq experiment was performed in duplicate, and ChIP-seq libraries were sequenced on either Illumina Hiseq 2000 or Nextseq 500 machines. All ChIP-seq data were mapped to hg19 using BWA (default parameters), and peaks were called using Sole-Search as previously described [8]. All ChIP-seq data were deposited in GEO (accession number GSE78913). Access to other publicly available ChIP-seq datasets used in this study can be found in Additional file 2: Table S1.

### FAIRE-seq

FAIRE assays were performed in MCF10A cells as previously described [5]. Two independent libraries were constructed and sequenced on Illumina Hi-Seq 2000. FAIRE-seq data were mapped to hg19 using BWA (default parameters), and peaks were called by using Sole-Search (TF parameter, alpha value: 0.001, fdr: 0.001). All FAIRE-seq data were also deposited in the accession number, GSE78913.

### siRNA knockdown, RT-qPCR, and RNA-seq

For transient knockdown, C42B cells were transfected in triplicate with 100 nM of siRNA oligonucleotides of human HOXC6 (Cat # L011871000005), DLX1 (Cat # L011871000005), or control (Cat # D0018101005) using SMART pool Dharmafect transfection reagent 3 (Dharmacon, Lafayette, CO, USA) for 72 h. RNA was extracted using Trizol reagent (Cat # 15596-026, Life technologies, Carlsbad, CA, USA) following its protocol. cDNA was synthesized using SuperScript^®^ VILO™ cDNA Synthesis Kit (Cat # 11754-050, Life technologies, Carlsbad, CA, USA). qPCR was performed on cDNA using SYBR Green (Cat # 172-5201, Bio-Rad, Hercules, CA, USA) with primers listed in Additional file 8: Table S7. RNA-seq libraries were made using KAPA Stranded mRNA-Seq Kit with KAPA mRNA Capture Beads (Cat # KK8420, Kapa Biosystems, Woburn, MA, USA). ERCC RNA Spike-In Mix (Cat # 4456740 Therma Fisher Scientific, Waltham, MA, USA) was added to each library for quality assessment. RNA-seq libraries were sequenced on Illumina Nextseq 500 with 75-bp single reads. To remove batch effects, matched controls and knockdown samples were prepared and sequenced at the same time. All RNA-seq data were deposited in the NCBI GEO accession number, GSE78913, and differentially expressed genes were selected by using the Gene Specific Algorithm from Partek^®^ Flow^®^ software using the upper quartile normalization method (Partek Inc., St. Louis, MO, USA). We used an FDR cutoff of 0.05 to select statistically significantly differently expressed genes. Differentially expressed genes with absolute fold change >1.5 were listed in Additional file 7: Table S6.

### Tracing enhancer networks using epigenetic traits (TENET)

To identify differentially methylated enhancers, the genomic coordinates of enhancers identified by REMC and ENCODE for 98 tissues or cell lines plus H3K27Ac ChIP-seq peaks from breast, prostate, and kidney cells were used. We then narrowed the regions by intersecting with the set of ENCODE Master DNaseI-seq peaks from 125 tissues or cell lines and DNaseI/FAIRE/NOMe-seq peaks from breast, prostate, and kidney cells (Additional file 2: Table S1). Only distal regulatory elements were used; these were located greater than 1.5 kb from a known TSS and identified using GENCODE v19 [36]. DNA methylation HM450 data and RNA-seq data of breast, prostate, and kidney tissues were downloaded from the TCGA data portal (https://tcga-data.nci.nih.gov/tcga/) and used to identify differentially methylated enhancer probes and their associated genes by developing an approach called TENET (freely available to download at http://farnhamlab.com/software). Importantly, this method can predict enhancer:gene links using only DNA methylation and RNA-seq data, which is easily obtainable from frozen tissues. In step 1 of TENET, differentially methylated enhancers in tissue samples are identified, adjusting for tumor purity. In steps 2–4 of TENET, relationships between enhancer activity and gene expression levels are investigated genome-wide. TENET was designed to detect enhancer activity changes and enhancer:gene links that are specific to tumor subgroups. The ability of TENET to annotate enhancer:gene links genome-wide also allows the identification of a set of key TFs for each tumor type. All enhancer to gene links found can be summarized and visualized using the tools in step 5 of TENET, which creates tables annotating enhancer to gene link states of each sample, statistic tables, histograms, scatterplots, circos plots, and genome browser tracks. A detailed explanation of the TENET, including information on installation, parameter settings, and statistical methods, is available in Additional file 1: Supplementary Methods.

### Comparison of TF ChIP-seq with TENET results

We obtained GATA3, FOXA1, and ESR1 ChIP-seq from ENCODE (Additional file 2: Table S1) and then tested whether the TF peaks were found within ±100 bp of each probe. Fisher exact tests were conducted between groups, and *p* values were adjusted using Benjamini–Hochberg method.

### Whole genome bisulfite sequencing (WGBS)

We used level 3 data of WGBS of breast invasive carcinoma (BRCA), colon adenocarcinoma (COAD), uterine corpus endometrioid carcinoma (UCEC), lung adenocarcinoma (LUAD), lung squamous cell carcinoma (LUSC), rectum adenocarcinoma (READ), and stomach adenocarcinoma (STAD) from the TCGA data portal (https://tcga-data.nci.nih.gov/tcga/), and we visualized these datasets using the Integrative Genomics Viewer (IGV) (https://www.broadinstitute.org/software/igv/).

### Heatmap of E^T^:G links for prostate tumors

Using a binary file of E^T^:G^+^ links found by TENET for PRAD in step 5, unsupervised clustering was performed using a binary method for distance matrix computation and Ward’s method for hierarchical clustering. On the top of the heatmap, previously defined genomic alternations commonly found in prostate tumors and Gleason scores of the tumors are indicated [14]. The images of prostate tumor tissues submitted to TCGA were reviewed according to the American Joint Committee on Cancer (AJCC) and assigned a Gleason score, which describes how dangerous a prostate tumor is in terms of how likely it is to metastasize; the higher Gleason score, the more likely that tumor will grow and spread quickly.

### Gene ontology (GO) and GSEA analysis

To identify E^T^:G^+^ links found common across prostate tumors, the resulting dendrogram from the unsupervised clustering of E^T^:G^+^ links was cut (*k* = 5), and 1075 links between 115 unique genes and 102 unique enhancer probes were found (cluster 1 of the Fig. 7). The 115 genes were analyzed for enrichment in particular GO categories using the TopGO program [37]. A Fisher exact test was performed, and an adjusted *p* value cutoff 0.05 was used to select statistically significantly enriched GO categories. For enrichment analysis of genes differentially expressed in knockdown experiments, genes with FC cutoff 1.2 and FDR cutoff, 0.05 were selected, and the above Fisher exact tests were used to determine enriched GO categories. The same differentially expressed genes were used to identify enriched gene sets using the GSEA (Gene Set Enrichment Analysis) tool [38]. Hypergeometric test was used to calculate *p* value, and false discovery rate (*q*-value) <0.05 was used to select significantly enriched gene sets.

### Motif analysis for TF-linked enhancers

To discover de novo motifs enriched in the enhancers linked to a TF, we collected sequences of 100-bp windows of the CpG probes and used MEME version 4.10.1 [25] with a minimum motif width of 6 and a maximum motif width of 12, scanning both strands of DNA sequences. To provide a stringent analysis, we reported de novo motifs found at enhancer probes using *E*-value cutoff, 0.0001, that were found in >50% of the TF-linked enhancers; see Additional file 9: Table S8. Two motifs (motif 1 and motif 2) were found to be enriched in the 183 enhancers linked to ZNF395 with an *E*-value cutoff, 0.0001. FIMO version 4.10.1 [39] was used to scan distal (>1500 bp from a TSS) ZNF395 ChIP-seq peaks in K562 cells expressing eGFP-ZNF395 (*n* = 7767), non-ZNF395 linked enhancers (*n* = 2352) from TENET in KIRC, and distal NDRs defined using the ENCODE DNaseI master sites for 125 cell types (*n* = 2391,038) for the presence of motif 1 and motif 2; only loci with a match *p* value <1 × 10^−4^ were counted (Fig. 8c).

### Survival analysis

A Kaplan–Meier survival analysis was used to estimate the association of *ZNF395* expression with the survival of kidney cancer patients. Overall survival was calculated using an R package, survival version 2.38 (http://CRAN.R-project.org/package=survival), with the date of initial diagnosis of cancer and disease-specific death or months to last follow-up for patients who are alive. After grouping kidney tumor samples with low (below mean) and high (above mean) *ZNF395* expression, a log rank test was performed.

## Additional files

**Additional file 1.** **Supplementary Methods**, **Figure S1.** Workflow of TENET. **Figure S2.** Schematic diagrams explaining the methodology of TENET. **Figure S3.** Comparison of enhancer probes in the three tumor types. **Figure S4.** Identification of genes linked to differentially methylated enhancers using TENET. **Figure S5.** Examples of enhancer probe:gene links identified by TENET. **Figure S6.** Distribution of enhancer probe:gene links on the same chromosome. **Figure S7.** Histograms of TENET-identified enhancer:gene links (centered on genes). **Figure S8.** Histograms of TENET-identified enhancer:gene links (centered on enhancers). **Figure S9.** Survival curves of ZNF395 in KIRC. **Figure S10.** Example of a 3-layer (TF-enhancer-gene) network. and **Supplementary References**.

**Additional file 2: Table S1.** Next generation sequencing data used for analyses.

**Additional file 3: Table S2.** TENET parameter settings used for BRCA, PRAD, and KIRC.

**Additional file 4: Table S3.** Summary of TENET results for all enhancer:gene links.

**Additional file 5: Table S4.** List of E^T^:G^+^ links found by TENET (a) BRCA, (b) PRAD, and (c) KIRC.

**Additional file 6: Table S5.** List of TFs found from TENET E^T^:G^+^ links for BRCA, PRAD, and KIRC.

**Additional file 7: Table S6.** List of the most statistically significantly differentially expressed genes after siRNA experiments for *HOXC6* and *DLX1*.

**Additional file 8: Table S7.** Oligonucleotides sequences used for RT-qPCR after siRNA experiments.

**Additional file 9: Table S8.** List of de novo motifs enriched in TFs found from TENET E^T^:G^+^ links for KIRC.

## Abbreviations

BRCA: breast invasive carcinoma
ChIP: chromatin immunoprecipitation
ENCODE: encyclopedia of DNA elements
FAIRE: formaldehyde-assisted isolation of regulatory elements
KIRC: kidney renal clear cell carcinoma
NOMe-seq: nucleosome occupancy and methylome sequencing
PRAD: prostate adenocarcinoma
REMC: roadmap epigenome mapping consortium
TCGA: the cancer genome atlas
TENET: tracing enhancer networks using epigenetic traits
TF: transcription factor
TSS: transcription start site
