# Supplementary material for: Identification of activated enhancers and linked transcription factors in breast, prostate, and kidney tumors by tracing enhancer networks using epigenetic traits
Source: Epigenetics Chromatin. 2016 Nov 9;9:50. doi: 10.1186/s13072-016-0102-4 (PMC5103450; doi:10.1186/s13072-016-0102-4)
Supplement: Supplementary file 1 — Additional file 1. Supplementary Methods, Figure S1. Workflow of TENET. Figure S2. Schematic diagrams explaining the methodology of TENET. Figure S3. Comparison of enhancer probes in the three tumor types. Figure S4. Identification of genes linked to differentially methylated enhancers using TENET. Figure S5. Examples of enhancer probe:gene links identified by TENET. Figure S6. Distribution of enhancer probe:gene links on the same chromosome. Figure S7. Histograms of TENET-identified enhancer:gene links (centered on genes). Figure S8. Histograms of TENET-identified enhancer:gene links (centered on enhancers). Figure S9. Survival curves of ZNF395 in KIRC. Figure S10. Example of a 3-layer (TF-enhancer-gene) network. and Supplementary References. [file 13072_2016_102_MOESM1_ESM.pdf]

Additional File 1, including Supplementary Methods, Supplementary Figures, and Supplementary References.

1 Supplementary Methods

Tracing Enhancer Networks using Epigenetic Traits (TENET)

To facilitate understanding of enhancer networks, we have developed a method called Tracing Enhancer Networks using Epigenetic Traits (TENET), which not only identifies active and inactive enhancers in each sample but also investigates enhancer to gene links genome-wide (**Additional file 1: Figure S1**). In step 1 of TENET, differentially methylated enhancers in tissue samples are identified adjusting tumor purity. In step 2 through 4 of TENET, relationships between enhancer activity and gene expression levels are investigated genome-wide. Previously, we tested relationships between an enhancer and 20 genes adjacent to an enhancer in the ELMER package [1]. However, some enhancers may regulate genes that are far away in a genomic coordinate distance, but proximal in a three dimensional space. Additionally, studying relationships between enhancers and genes in a genome-wide manner is especially useful for the analysis of TFs. We recognize that testing all interactions between enhancers and genes can be difficult due to multiple comparisons and sample size effects on statistics. However, TENET can be used to compare the activity of an enhancer to the expression of all genes and the expression of a gene to the activity state of all enhancers in step 2 through 4 by narrowing down the comparisons. We designed TENET to first calculate z scores between an enhancer to all genes after grouping samples to unmethylated and methylated groups. Then, using the calculated z scores, p values are permutated between a gene to all enhancers, and links with statistically significant association are chosen. Lastly, to optimize the enhancer to gene link selection, Wilcoxon rank sum test is performed between samples with an active enhancer status and samples with inactive enhancer status (**Additional file 1: Figure S2**). The TENET program has the option to identify all genes that are either positively or negatively associated with an enhancer (**Additional file 1: Figure S4**). All enhancer to gene links found may be summarized and visualized using the tools in step 5 of TENET, which creates tables annotating enhancer to gene link states of each sample, statistic tables, histograms, scatterplots, circosplots, and genome browser tracks. Importantly, we designed TENET for users to be able to alter parameters and even run only specific steps in the program.

Installation of the TENET program
TENET.tar.gz is publicly available to download with detailed installation explanation in http://farnhamlab.com/software

Step 0: Obtain DNA methylation and gene expression data
Level 3 DNA methylation HM450 data and RNA-seq data of tumor and normal tissue samples for PRAD, BRCA, and KIRC were downloaded from the TCGA data portal [2-4] (prostate adenocarcinoma (PRAD): 333 tumors, 19 normals; breast invasive carcinoma (BRCA): 641 tumors, 66 normals; kidney renal clear cell carcinoma (KIRC): 318 tumors, 24 normals). As we have described in previous TCGA manuscripts [2-4], gene expression levels were normalized using the upper quartile normalization method for RSEM count estimates and log transformed (log2(RSEM+1)) values were used for downstream analyses. For DNA methylation, background-corrected intensities of methylated (M) and unmethylated (U) calls and detection P-values of each probe were measured. After monitoring technical variations among batches of samples along with control cell line technical replicates, β values (M/(M+U)) were calculated for probes but excluding those probes with the following characteristics: 1) probes that have a common SNP (dbSNP build 135, minor allele frequency >1%) within 10 bp of the interrogated CpG site, 2) probes that are located within 15bp of a repetitive element (Repeat Masker and Tandem Repeats Finder from UCSC hg19, Feb 2009), 3) probes that are aligned to multiple sites on human genome (UCSC hg19, Feb 2009), and 4) probes that have detection P values greater than 0.05 for a specific data point.

Step 1: Identification of differentially methylated enhancer probes (see Additional file 1: Figure S2a).
We used the genomic coordinates of enhancers identified by the Roadmap Epigenomics Mapping Consortium (REMC) and the Encyclopedia of DNA Elements (ENCODE) Project for 98 tissues or cell lines, collected from the ELMER package [1] (parameter, elmerENH), plus genomic coordinates of H3K27Ac ChIP-seq peaks from several cancer cell lines and normal cells for BRCA, PRAD, and KIRC (parameter, extENH) (**Additional file 2: Table S1**). We then selected the subset of these regulatory elements that are located greater than 1.5kb (parameter, udist, ddist) from a known transcription state site (TSS), as defined using GENCODE v19 from the ELMER package [1]. We further narrowed the regions by intersecting with the set of ENCODE Master DNaseI-seq peaks from 125 tissues or cell lines (parameter, encodeNDR) or DNaseI-seq/FAIRE-seq/NOME-seq peaks of corresponding cell types (parameter, extNDR) (**Additional file 2: Table S1**). Using these criteria, we identified 64231, 85977, and 63996 probes on the HM450 array that overlapped the defined enhancer regions for PRAD, BRCA, and KIRC, respectively. Then, we identified the subset of these probes that showed changes in DNA methylation levels in normal vs. tumor tissues and were not affected by tumor purity (mainly due to leukocyte infiltration (parameter, leuk)). We identified 4471 (PRAD), 3033 (BRCA), and 5172 (KIRC) probes that were unmethylated in both normal and tumor samples (mean of β_{normal}<0.2 (parameter, unmethcutoff), mean of β_{tumor}<0.2, β≥0.2 in less than 5 tumors (parameter, minTumor)); we identified

10531 (PRAD), 5364 (BRCA), and 6657 (KIRC) probes that were methylated in both normal and tumor samples (mean of β_{normal}>0.8 (parameter, methcutoff), mean of β_{tumor}>0.8, β≤0.8 in less than 5 tumors (parameter, minTumor)); we identified 4092 (PRAD), 7522 (BRCA), and 3910 (KIRC) probes that were hypermethylated in tumors as compared to normal tissues (mean of $\beta_{leukocyte}$<0.2 (parameter, unmethcutoff), mean of β_{normal}<0.2, β_{tumor}>0.3 (parameter, hypercutoff) in more than 5 tumors (parameter, minTumor)), and we identified 6251 (PRAD), 19882 (BRCA), and 10730 (KIRC) probes that were hypomethylated in tumors as compared to normal tissues (mean of $\beta_{leukocyte}$>0.8 (parameter, methcutoff), mean of β_{normal}>0.8, β<0.7 (parameter, hypocutoff) in more than 5 tumors (parameter, minTumor)). In the TENET program, DNA methylation datasets of leukocytes, smooth muscles, and fibroblasts are included and there is an option for users to include additional DNA methylation datasets of other cell types to identify differentially methylated enhancer regions not affected by purity. For this study, we set hypomethylation and hypermethylation cut-offs based on the distribution of tumor cellularity among the tumor samples we used: most TCGA tumor samples had > 20% tumor purity, and dichotomization of data with β > 0.3 for the hypermethylation and β < 0.7 for the hypomethylation was used to alleviate the influence of variable levels of tumor purity. We designed TENET such that it can detect changes that occur in a small number of tumors (parameter, minTumor), using absolute DNA methylation differences between tumor and normal samples (**Additional file 1: Figure S2**). In order to identify links found in about 1% of tumor samples, we used 5 tumor samples as a threshold. TENET is designed for users to set their own thresholds for beta values and the number of tumor samples, depending on individual datasets. In addition, TENET can investigate DNA methylation and gene expression relationships (see below) among cases only, allowing the detection of enhancer to gene links uniquely found in a small subgroup of cases (i.e. tumors, in this study).

Step 2: Identification of differentially methylated enhancer probe to gene links using Z scores (see Additional file 1: Figure S2b, left panels and right top panel)
The steps to identify statistically significantly associated enhancer probe-gene links are: 1) to alleviate the tumor purity effect on DNA methylation analysis, for each enhancer probe we grouped the tumor samples into a set of unmethylated tumors (for hypermeth: β<0.3 (parameter, hypercutoff), for hypometh: β<0.7 (parameter, hypocutoff)) and a set of methylated tumors (for hypermeth: β>0.3, for hypometh: β>0.7), 2) a Z score was measured by first subtracting the mean of gene expression levels in the unmethylated tumors from the mean of gene expression levels in the methylated tumors then dividing by the standard deviation of gene expression levels in the unmethylated tumors, 3) enhancer probe to gene links with a Z score > |±1.645|, one tailed test critical value for 95% confidence level, were selected (parameter, Zcutoff) (a Z score < -1.645 was used for positive enhancer-gene expression relationships (E^N:G^+ or E^T:G^+) and a Z score > 1.645 was used for negative enhancer gene-expression relationships (E^N:G^- or E^T:G^-)). When the parameter, usecaseonly is FALSE, all samples were used to group into either a set of unmethylated samples, or methylated samples depending on DNA methylation levels, and Z scores were calculated.

Step 3: Identification of significant enhancer probe to gene links using permutation (see Additional file 1: Figure S2b, right bottom panel)
To identify statistically significant links, a permutation test was performed with all of the other hyper or hypomethylated probes for each gene from the links found in step 2. An empirical p value cut-off, 0.05 (parameter, permutation.cutoff) was used to further refine the set of enhancer probes linked to expression of specific genes in this study.

Step 4: Optimize selection of enhancer probe to gene links (see Additional file 1: Figure S2c)
To ensure the enhancer probe to gene links have statistically significant changes in gene expression levels between the hyper or hypomethylated tumors and the normal tissue samples, a Wilcoxon rank sum test was used to test the null hypothesis that overall gene expression in hyper or hypomethylated tumor samples was different from that in normal samples (adj. p value <0.05 (parameter, adj.pval.cutoff); genes with expression levels higher in normal samples than in tumor samples were selected for categories of enhancer probe:gene links, E^N:G^+ or E^T:G^-, and genes with expression levels higher in tumor samples than in normal samples were selected for categories of enhancer probe:gene links, E^N:G^- or E^T:G^+; genes with low expression levels across samples (mean log2(RSEM+1) equal to 0) were removed. To select the enhancer probe and gene links found with a very substantial degree of change, only those enhancer probe-gene links having at least one tumor with a β>0.6 for hypermeth (parameter, hyper.stringency) and a β<0.4 for hypometh (parameter, hypo.stringency) were chosen for this study.

NOTE: Different cut-offs may work better for your own datasets (e.g. due to purity). Each parameter can be altered as the users prefer. We recommend users to try different cut-offs and confirm the relationship by visualizing in Step 5.

Step 5: Summarization and visualization of TENET results
To summarize the TENET results, all links including genomic coordinates of enhancer probes and TSS of associated genes, as well as p values, are written in *anno.txt files, and list of enhancer probes and genes are saved in *list.txt files.

To identify key transcription factors (using a 1,982 human transcription factor annotation from the ELMER package [1]), known tumor suppressors (using a set of 637 protein coding tumor suppressor genes from the TSGene resource [5], and known oncogenes (using a set of 537 known cancer genes from The Cancer Gene Census [6] for each tumor type,

enhancer/gene/TF/known oncogene/known tumor suppressor gene frequency tables (*freq.txt, *table.txt) and histograms (*hist.pdf, *barplot.pdf) are generated (e.g. parameter, hypoGposHistogram).

Enhancer probe to gene link states of each sample is listed in *states.table.txt files as a binary format (enhancer gene links status; yes=1, no=0) (e.g. parameter, hypoGposStates). The status of each individual enhancer probe:gene link in each case sample (i.e. tumor, in this study) is annotated using a hypomethylation cut-off and a hypermethylation cut-off for DNA methylation and a mean of each gene expression level in case samples (E^N:G^+ and E^T:G^-: lower than mean expression level of gene in case samples, E^T:G^+ and E^N:G^-: higher than mean expression level of gene in case samples).

For visualization, scatterplots between DNA methylation and gene expression levels (e.g. parameter, hypoGposScatter), circosplots between enhancers and genes (e.g. parameter, makeCircos4gene), and genome browser tracks are made (e.g. parameter, hypoGposTracks). In the scatterplots, empirical p values measured in step 3 and Spearman's correlation coefficients between DNA methylation and gene expression of each link are included for users to help determine TENET program's parameters of their datasets. In order to confirm that the enhancer probe:gene links identified by TENET were not confounded by other factors, complex scatterplots can be made with additional information of tumor samples. If tumor purity estimates, copy number variation (CNV), and somatic mutation (SM) datasets are available, complex scatterplots between DNA methylation and gene expression levels showing tumor purity as dot size and CNV and SM as dot shape (see **Additional file 1: Figure S2b**) can be generated (e.g. parameter, hypoGposCScatter). For PRAD TENET results, average DNA-based purity estimates and CNV and SM data were downloaded from the TCGA Firebrowse portal [2] and included to confirm that the enhancer probe-gene links identified by TENET were not confounded by other factors (**Additional file 1: Figure S2**).

NOTE: TENET is designed for users that each step can be run independently, so users can switch order of steps to run as they would like. For enhancer:gene links identification, we recommend users to run all of steps for linking (i.e. step 2, 3, and 4), reducing potential statistical bias; especially, when the parameter, minTumor is set with a very small number.

2 Supplementary Figures

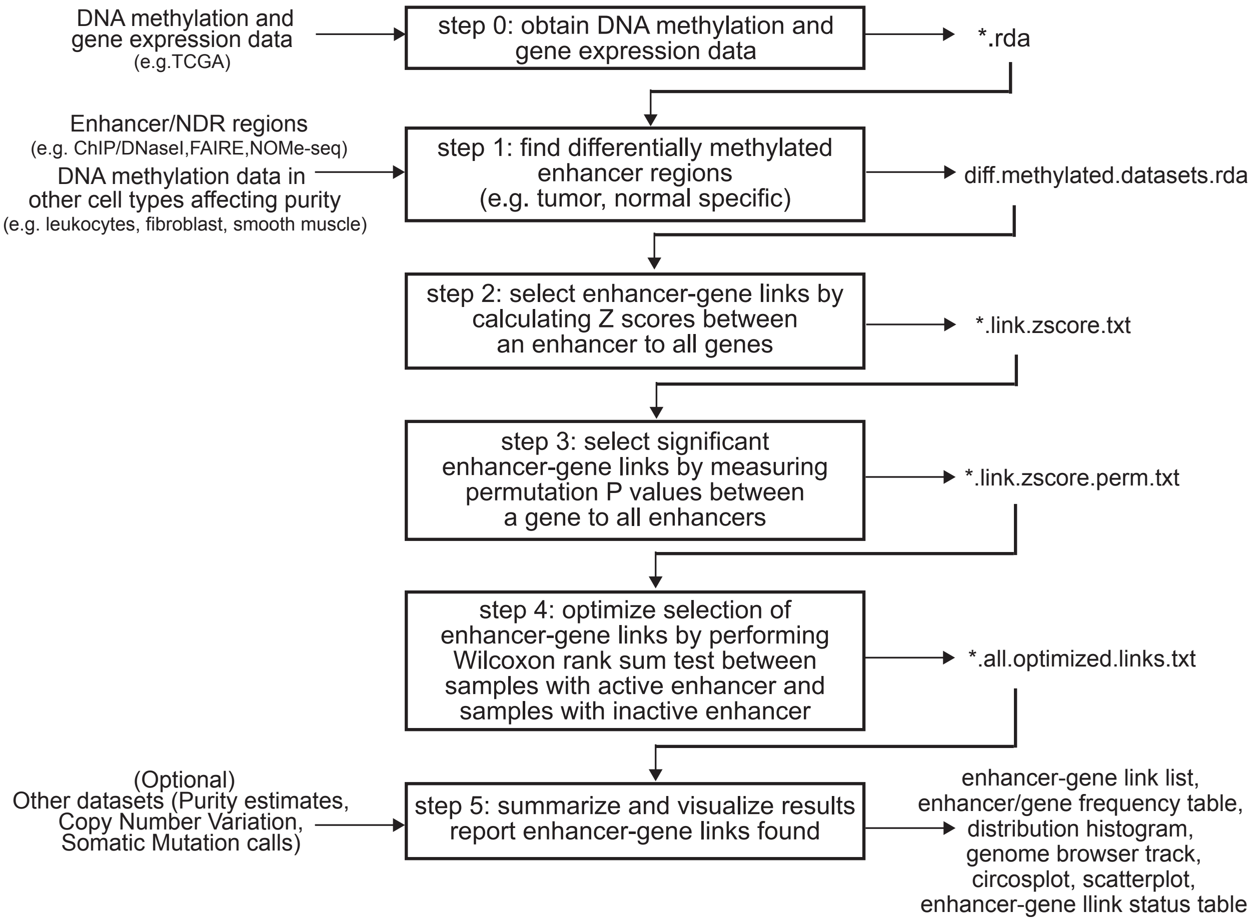

Figure S1. Workflow of TENET. Starting from obtaining datasets (step 0), TENET identifies differentially methylated enhancer regions (step 1), and selects enhancer-gene links using statistical methods (step2 through 4). The enhancer-gene links found by TENET can be visualized by making tables, histograms, scatterplots, circos plots, and genome browser tracks (step 5).

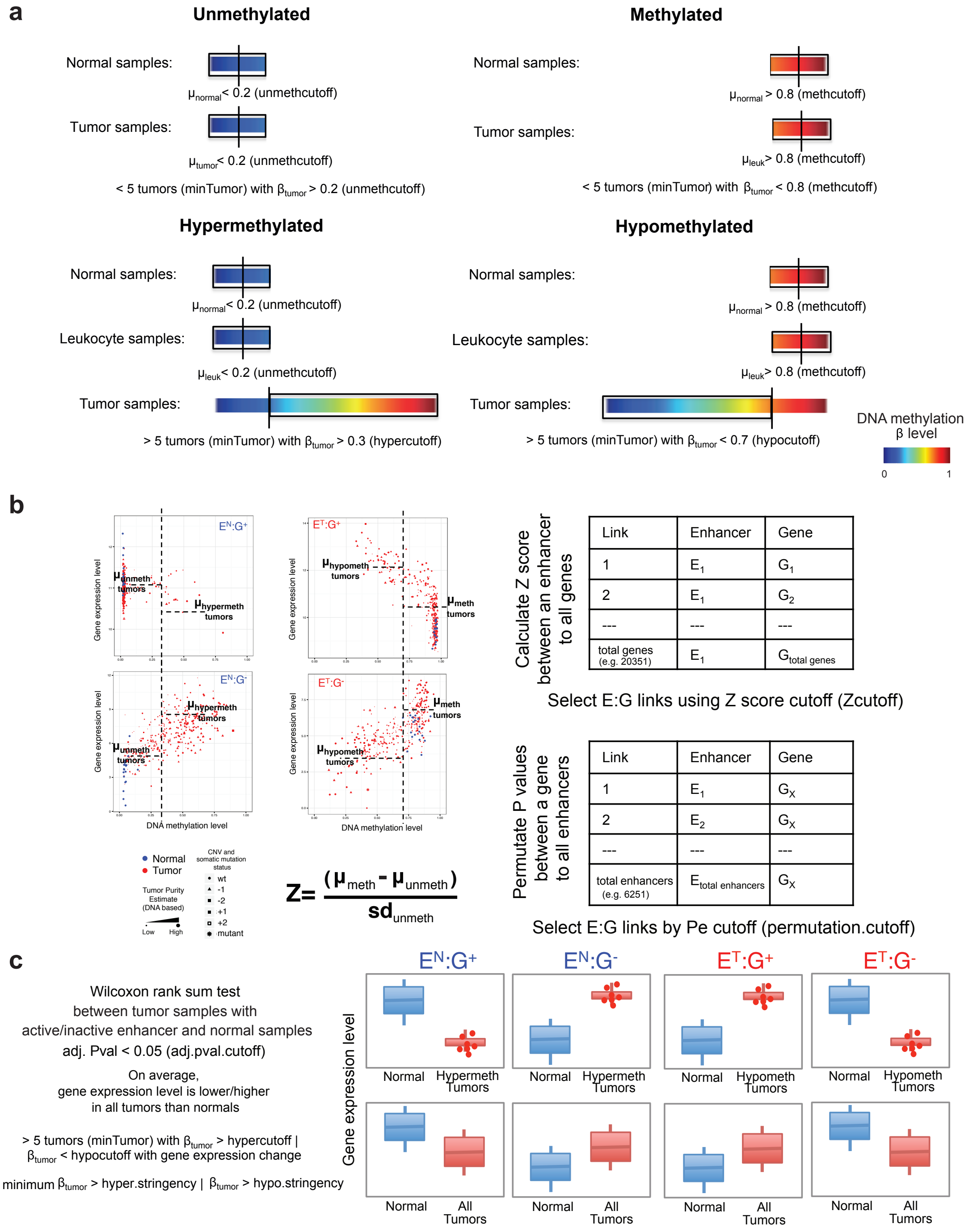

Link	Enhancer	Gene
1	E_1	G_1
2	E_1	G_2
---	---	---
total genes (e.g. 20351)	E_1	$G_{total\ genes}$

Link	Enhancer	Gene
1	E_1	G_X
2	E_2	G_X
---	---	---
total enhancers (e.g. 6251)	$E_{total\ enhancers}$	G_X

Figure S2. Schematic diagrams explaining the methodology of TENET. (**a**) Identification of differentially methylated enhancer regions (see Supplementary Methods for a detailed description of step 1 of TENET). (**b**) Shown on the left scatterplots are examples of E^N:G^+ and E^N:G^- links. Shown on the right scatterplots are examples of E^T:G^+ and E^T:G^- links. To further visually validate that the enhancer probe:gene expression relationship is not confounded by tumor purity, the average DNA-based tumor purity estimates, measured by ABSOLUTE [7] and CLONET [8], are indicated by the size of each dot in the scatter plots in panels A and B. Additionally, somatic mutation information measured by MutSig2CV [9], and copy number variation of each gene analyzed by GISTIC2 calls [10] are reflected as dot shapes to investigate any effects of genetic alteration on gene expression [2]. Z score was calculated between an enhancer to all genes (e.g. n=20,531 for TCGA Level 3 RNA-seq datasets) in step 2 (top right). Using z scores, empirical p values were permutated between the gene selected in step 2 to all other hypo or hypermethylated enhancers (e.g. n=6,251 for hypomethylated enhancer probes in PRAD) in step 3 (bottom right). (**c**) To further optimize selection of enhancer:gene links, gene expression levels were compared between tumor and normal samples in step 4.

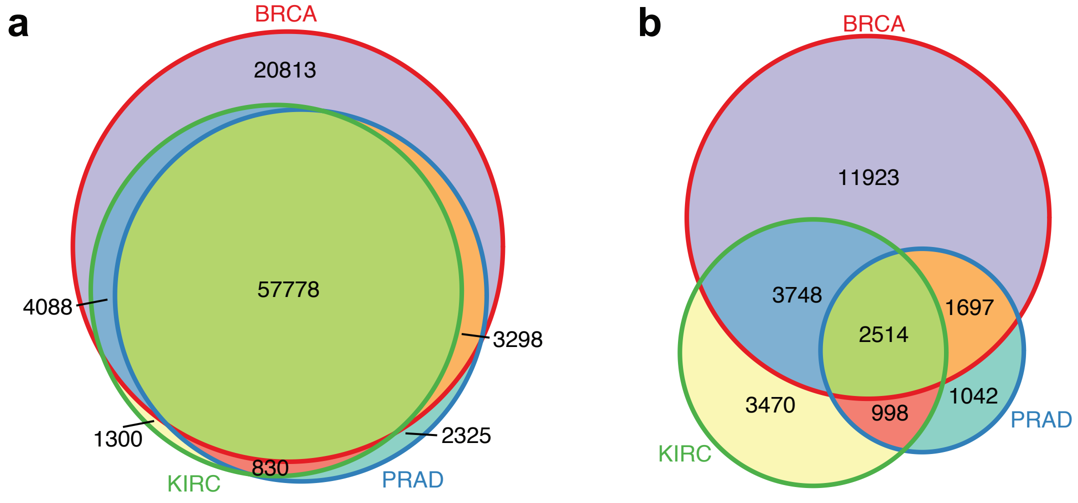

Figure S3. Comparison of enhancer probes in the three tumor types. (**a**) Comparison of all enhancer probes studied in the three tumor types, including inactive and active enhancers (BRCA: 85,977, PRAD: 64,231, KIRC: 63,996). (**b**) Comparison of hypomethylated enhancer probes (corresponding to enhancers gained in tumors) from three tumor types (BRCA: 19,882, PRAD: 6,251, KIRC: 10,730).

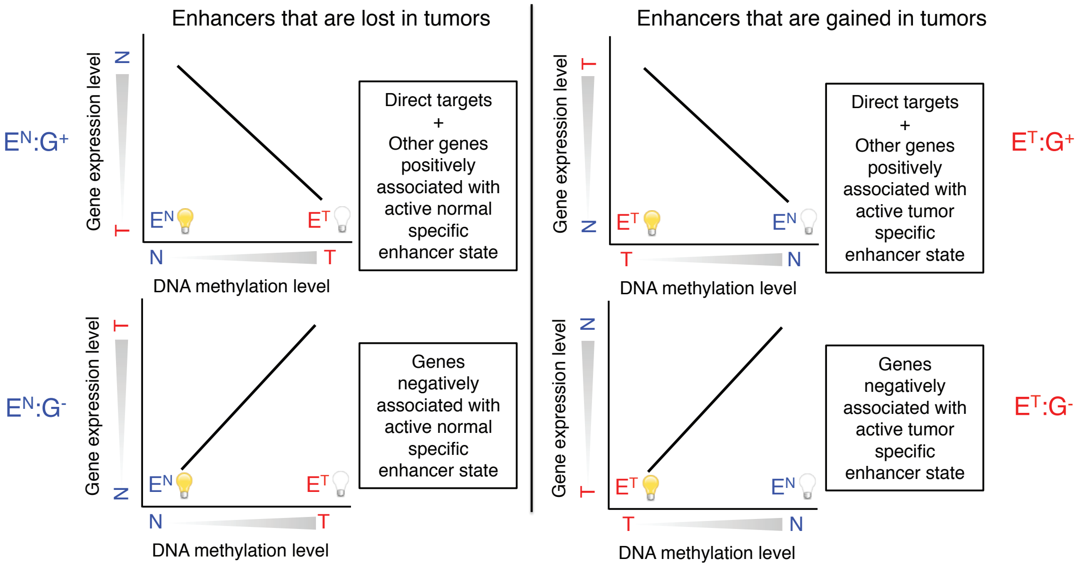

Figure S4. Identification of genes linked to differentially methylated enhancers using TENET. Schematic diagrams explaining TENET enhancer:gene links. **Left panels** represent analysis of normal-specific enhancers (E^N) that lose activity in tumors. **Right panels** represent tumor-specific enhancers (E^T) that gain activity in tumors. **Top**: genes directly targeted by enhancers and other genes positively associated with an active enhancer state (Left: E^N:G^+, Right: E^T:G^+). **Bottom**: genes negatively associated with active enhancer states (Left: E^N:G^-, Right: E^T:G^-). X axis: DNA methylation level of the enhancer probe. Y axis: gene expression level. Light bulbs indicate enhancer states (E^T, E^N) for each link (active – yellow, inactive – white).

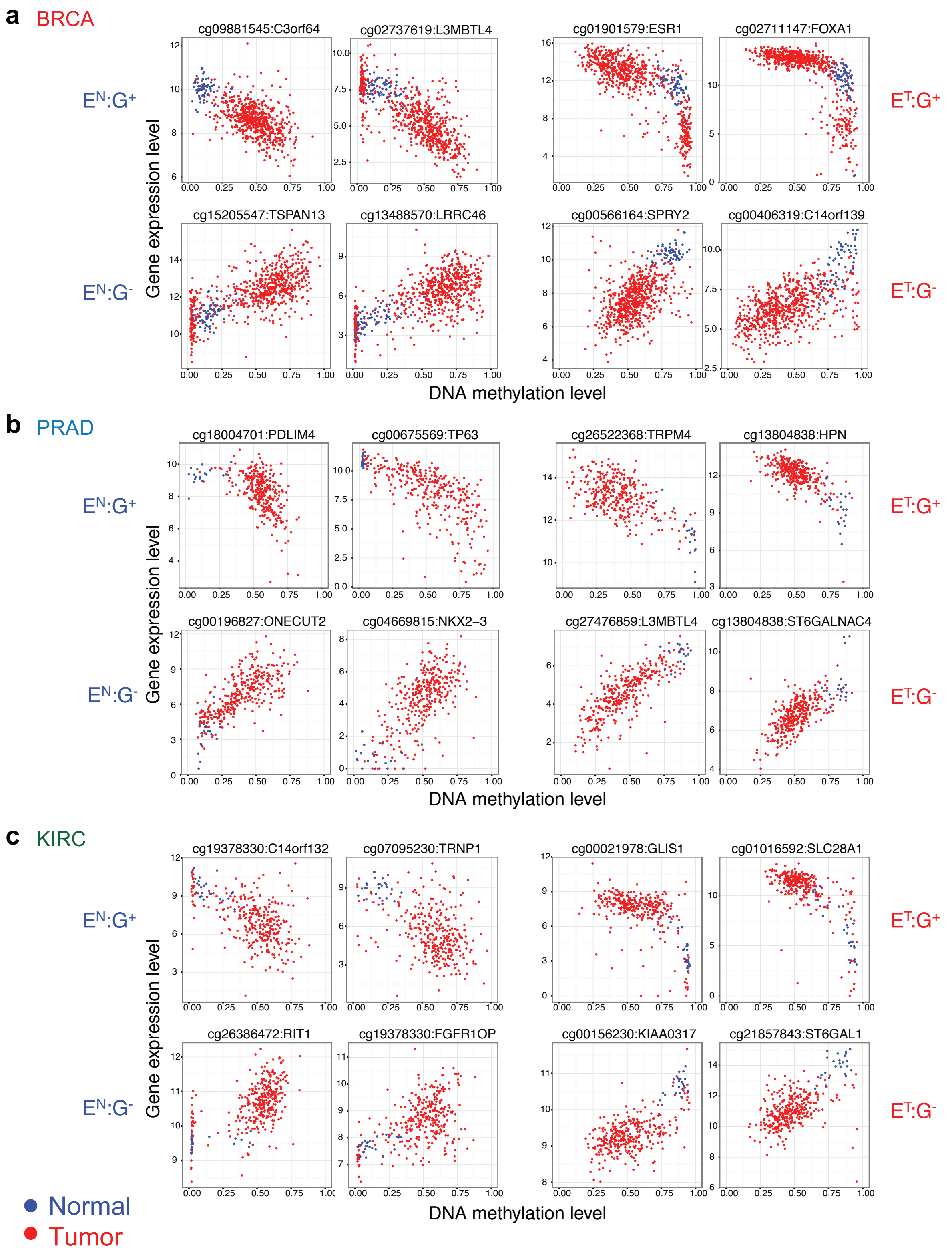

Figure S5. Examples of enhancer probe:gene links identified by TENET. (**a**) PRAD, (**b**) BRCA, and (**c**) KIRC. Top left: E^N:G^+. Bottom left: E^N:G^-. Top right: E^T:G^+. Bottom right: E^T:G^-; for descriptions of each category, see Figure S2 legend. X axis: DNA methylation level of the enhancer probe. Y axis: the gene expression level. Each sample is colored according to whether is normal (blue) or tumor (red). The enhancer probe and gene names for each link are indicated at the top of each scatterplot.

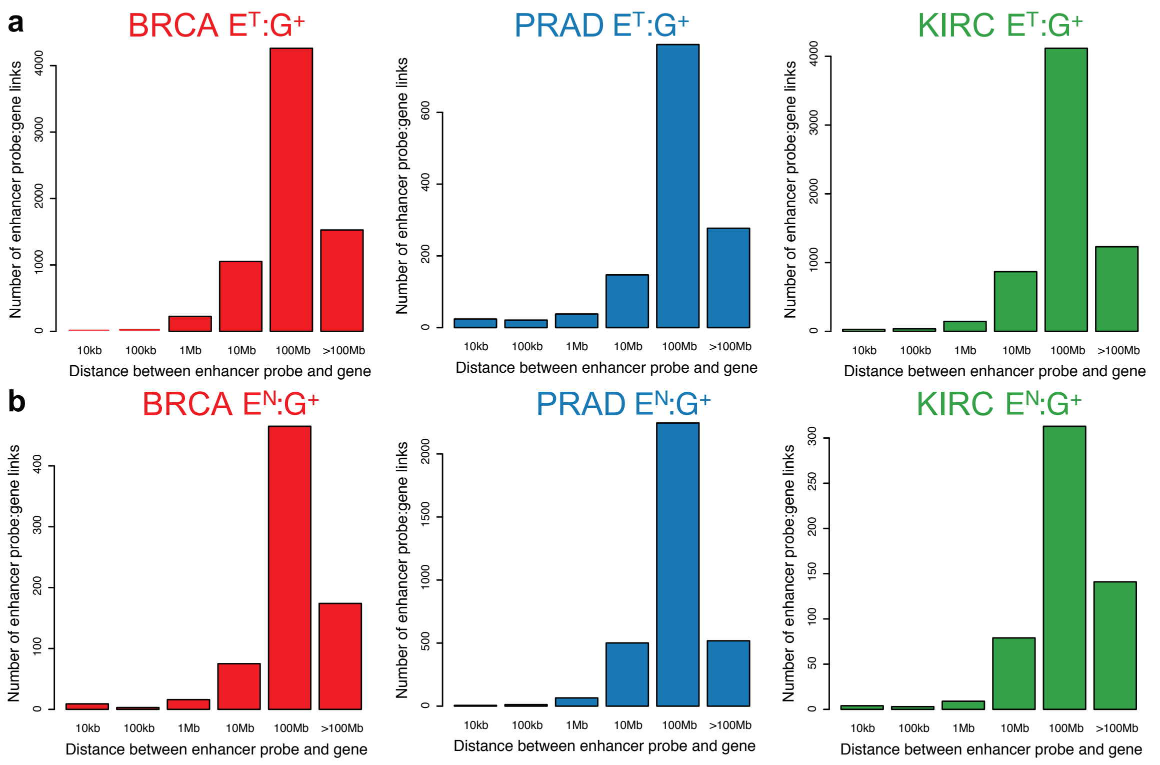

Figure S6. Distribution of enhancer probe:gene links on the same chromosome. Shown is the number of enhancer probe to gene links on the same chromosome by distance for the two different categories of enhancer probe:gene links [(**a**) E^T:G^+ and (**b**) E^N:G^+] in PRAD (left, red), BRCA (center, blue), and KIRC (right, green).

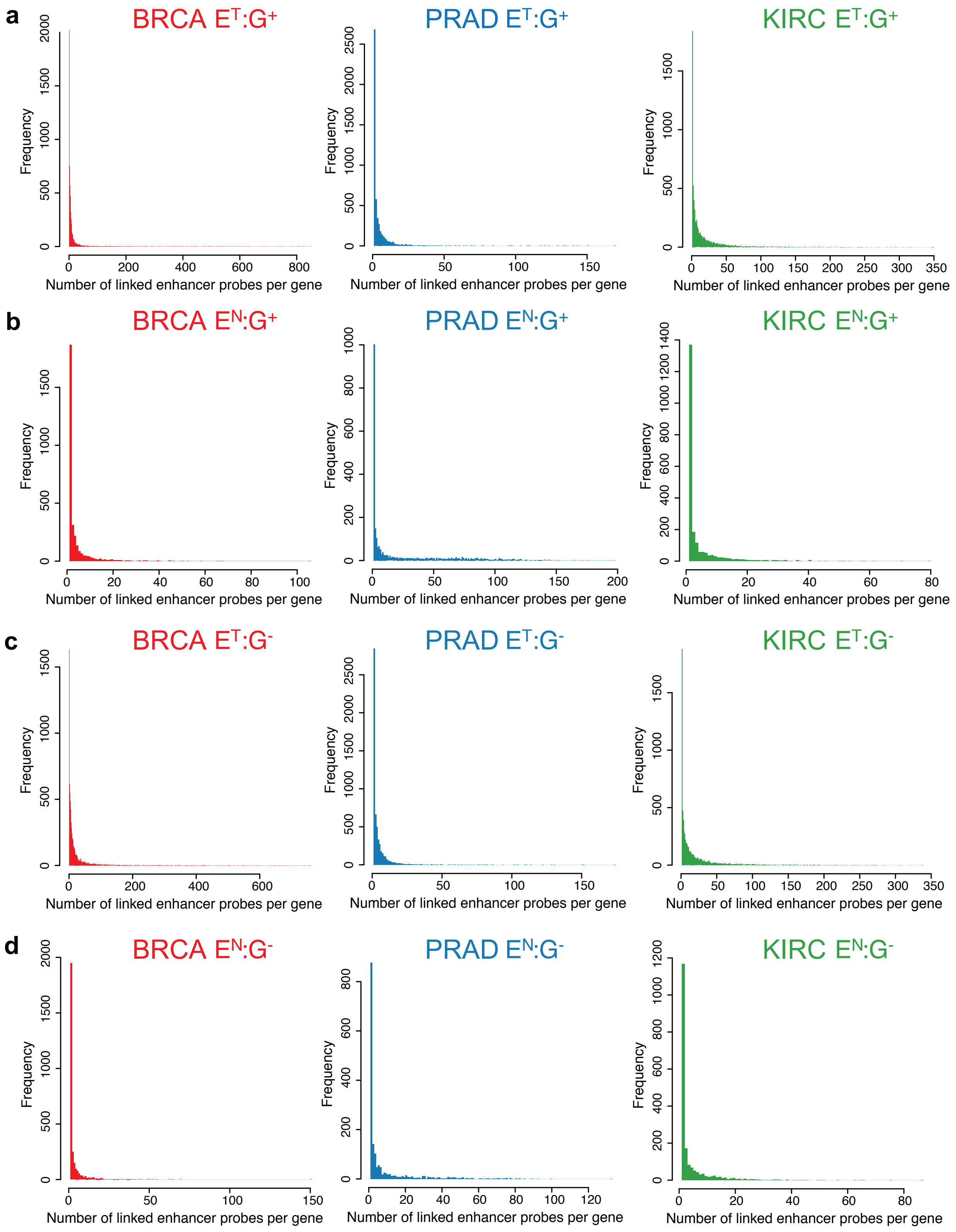

Figure S7. Histograms of TENET-identified enhancer:gene links (centered on genes). Shown is the number of linked enhancer probes per gene in the four different enhancer:gene link categories [(**a**) E^T:G^+, (**b**) E^N:G^+, (**c**) E^T:G^-, and (**d**) E^N:G^-] in PRAD (left, red), BRCA (center, blue), and KIRC (right, green).

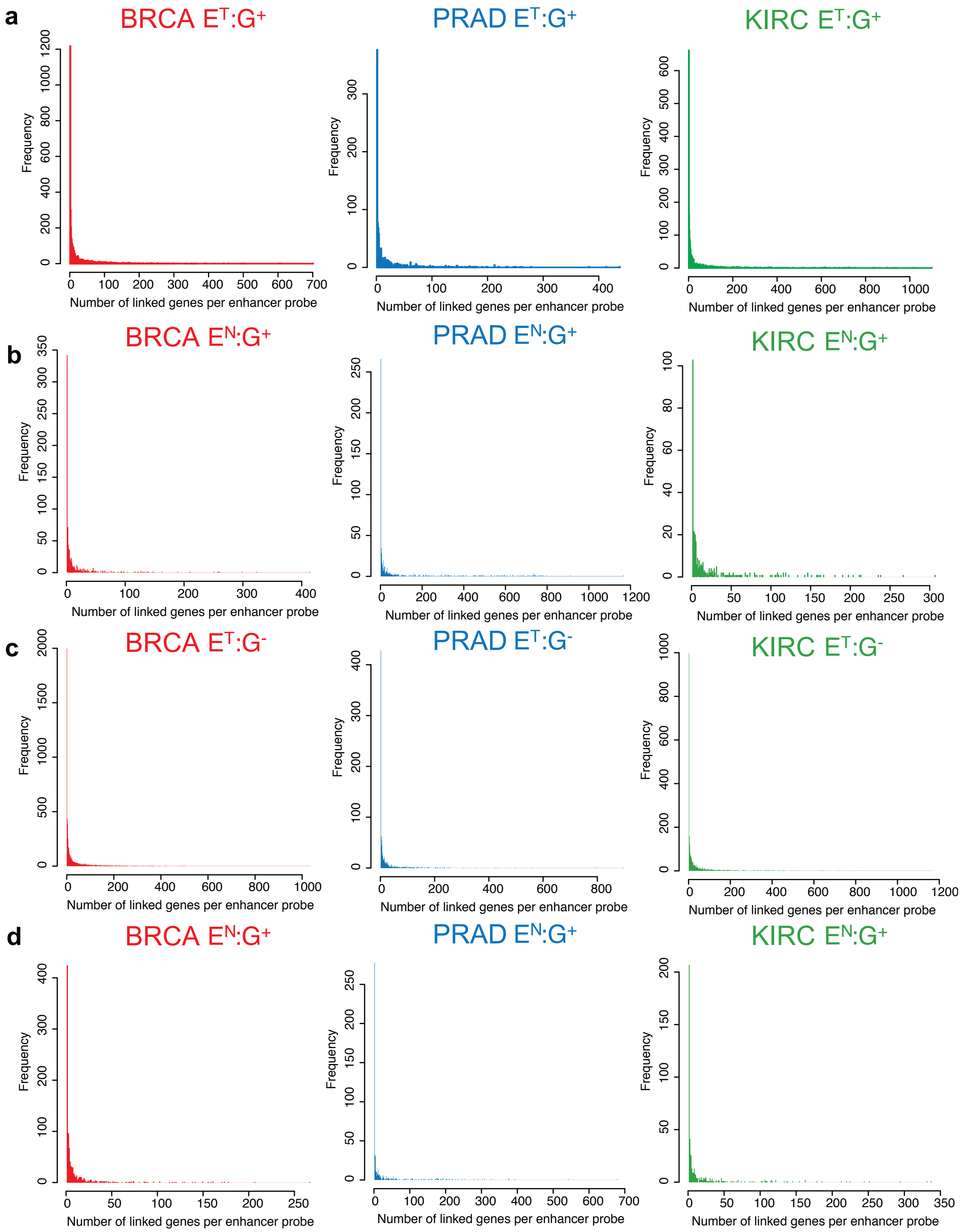

Figure S8. Histograms of TENET-identified enhancer:gene links (centered on enhancers). Shown is the number of linked genes per enhancer probe in the four different enhancer:gene link categories [(**a**) E^T:G^+, (**b**) E^N:G^+, (**c**) E^T:G^-, and (**d**) E^N:G^-] in PRAD (left, red), BRCA (center, blue), and KIRC (right, green).

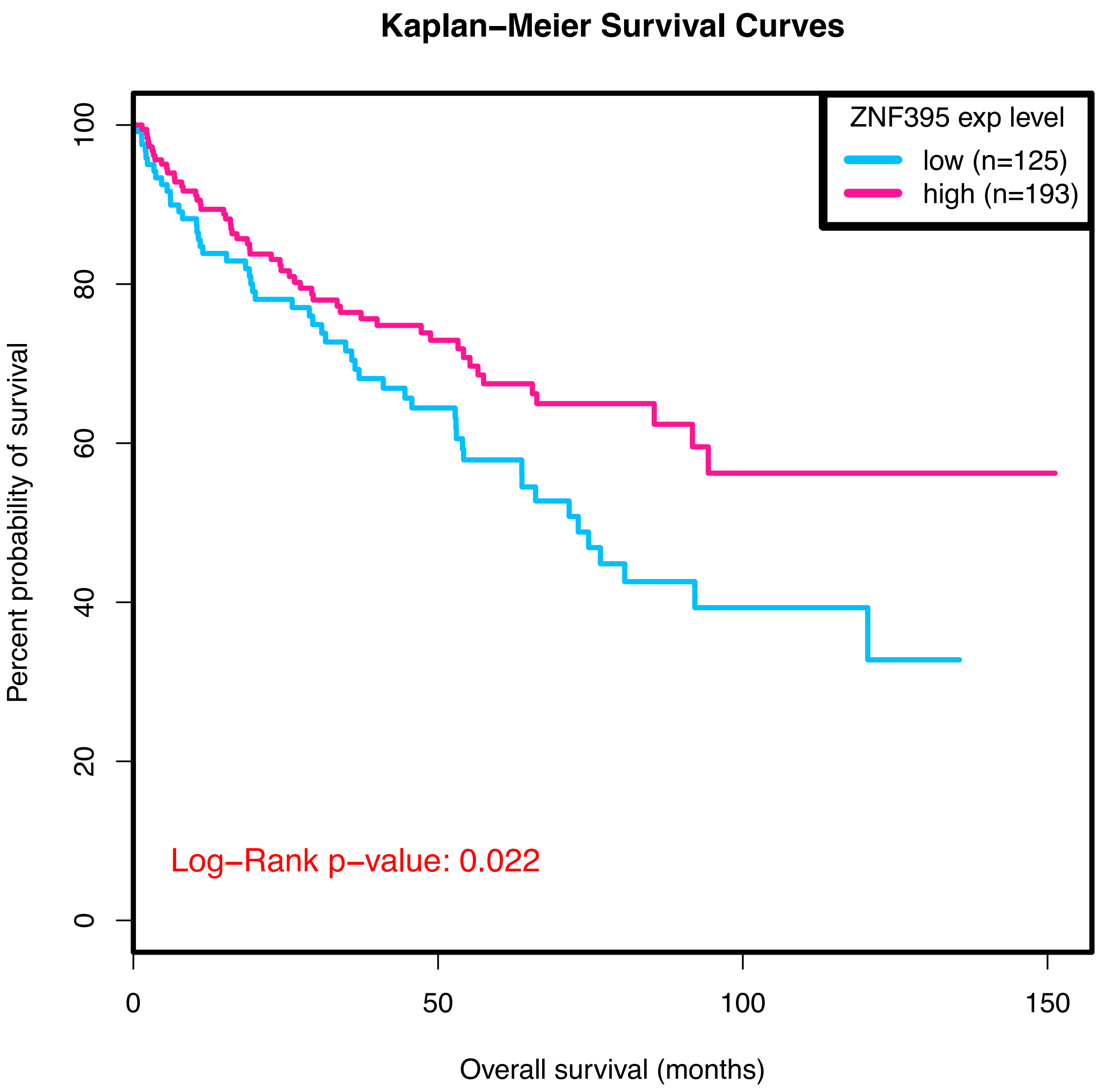

Figure S9. Survival curves of ZNF395 in KIRC. 318 kidney tumor tissue samples were grouped to low (blue) and high (pink) using the mean of *ZNF395* gene expression levels in tumors, and overall survival months were plotted.

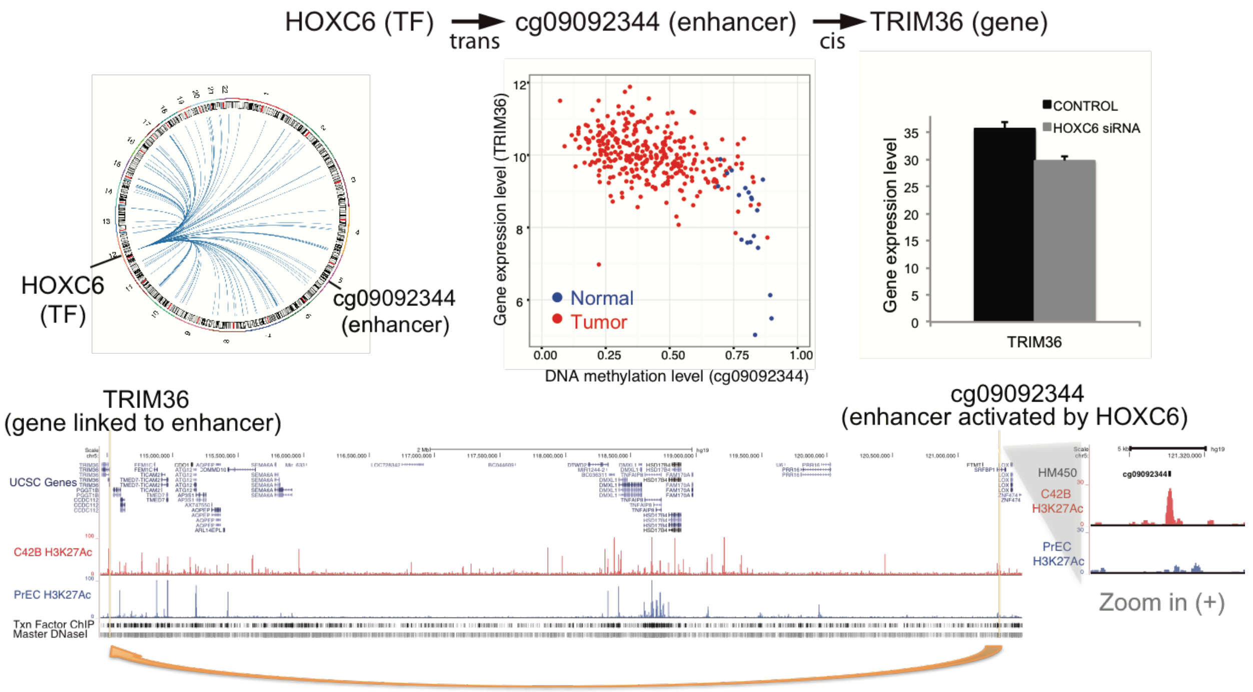

Figure S10. Example of 3-layer (TF-enhancer-gene) network. TF HOXC6 is linked to 121 prostate tumor-specific enhancers throughout the genome, including cg09092344 enhancer probe (top left). DNA methylation levels of the HOXC6-linked enhancer probe cg09092344 are correlated with expression level of TRIM36, which is located ~6Mb away from the enhancer probe (top center). TRIM36 is one of the genes showing significantly decreased expression in the HOXC6 siRNA knockdown experiment (top right). Genome browser screen shots show genomic coordinates, UCSC genes, H3K27Ac ChIP-seq tracks in C42B prostate tumor and normal prostate (PrEC) cells, the ENCODE layered ChIP-seq track for 161 TFs, and the ENCODE Master DNaseI hypersensitive site track for 125 cell types (bottom left). The enhancer probe cg09092344 and the TRIM36 gene are highlighted in yellow. A zoomed in view of the H3K27Ac ChIP-seq tracks near the enhancer probe cg09092344 is shown on the bottom right.

3 Supplementary References

1. Yao L, Shen H, Laird PW, Farnham PJ, Berman BP: **Inferring regulatory element landscapes and transcription factor networks from cancer methylomes**. *Genome biology* 2015, **16**:105.
2. Cancer Genome Atlas Research Network.: **The Molecular Taxonomy of Primary Prostate Cancer**. *Cell* 2015, **163**(4):1011-1025.
3. Ciriello G, Gatza ML, Beck AH, Wilkerson MD, Rhie SK, Pastore A, Zhang H, McLellan M, Yau C, Kandoth C *et al*: **Comprehensive Molecular Portraits of Invasive Lobular Breast Cancer**. *Cell* 2015, **163**(2):506-519.
4. Shinagare AB, Vikram R, Jaffe C, Akin O, Kirby J, Huang E, Freymann J, Sainani NI, Sadow CA, Bathala TK *et al*: **Radiogenomics of clear cell renal cell carcinoma: preliminary findings of The Cancer Genome Atlas-Renal Cell Carcinoma (TCGA-RCC) Imaging Research Group**. *Abdom Imaging* 2015, **40**(6):1684-1692.
5. Zhao M, Sun J, Zhao Z: **TSGene: a web resource for tumor suppressor genes**. *Nucleic Acids Res* 2013, **41**(Database issue):D970-976.
6. An O, Pendino V, D'Antonio M, Ratti E, Gentilini M, Ciccarelli FD: **NCG 4.0: the network of cancer genes in the era of massive mutational screenings of cancer genomes**. *Database (Oxford)* 2014, **2014**:bau015.
7. Carter SL, Cibulskis K, Helman E, McKenna A, Shen H, Zack T, Laird PW, Onofrio RC, Winckler W, Weir BA *et al*: **Absolute quantification of somatic DNA alterations in human cancer**. *Nat Biotechnol* 2012, **30**(5):413-421.
8. Prandi D, Baca SC, Romanel A, Barbieri CE, Mosquera JM, Fontugne J, Beltran H, Sboner A, Garraway LA, Rubin MA *et al*: **Unraveling the clonal hierarchy of somatic genomic aberrations**. *Genome biology* 2014, **15**(8):439.
9. Lawrence MS, Stojanov P, Polak P, Kryukov GV, Cibulskis K, Sivachenko A, Carter SL, Stewart C, Mermel CH, Roberts SA *et al*: **Mutational heterogeneity in cancer and the search for new cancer-associated genes**. *Nature* 2013, **499**(7457):214-218.
10. Mermel CH, Schumacher SE, Hill B, Meyerson ML, Beroukhim R, Getz G: **GISTIC2.0 facilitates sensitive and confident localization of the targets of focal somatic copy-number alteration in human cancers**. *Genome biology* 2011, **12**(4):R41.
