# Supplementary material for: Identification of activated enhancers and linked transcription factors in breast, prostate, and kidney tumors by tracing enhancer networks using epigenetic traits
Source: Epigenetics Chromatin. 2016 Nov 9;9:50. doi: 10.1186/s13072-016-0102-4 (PMC5103450; doi:10.1186/s13072-016-0102-4)
Supplement: Supplementary file 9 — Additional file 9: Table S8. List of de novo motifs enriched in TFs found from TENET ET:G+ links for KIRC. [file 13072_2016_102_MOESM9_ESM.pdf]

Table S8. List of de novo motifs enriched in TFs found from TENET E^T:G^+ links for KIRC

Motif ID	Motif profile	Linked TF	E-value	No. of loci with motif	Total no. of loci	Fraction of loci with motif
K1	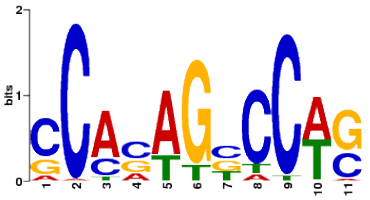	GLIS1	3.40E-07	134	222	0.60
K2	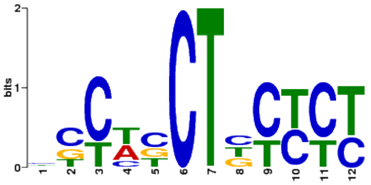	GLIS1	6.10E-10	138	222	0.62
K3	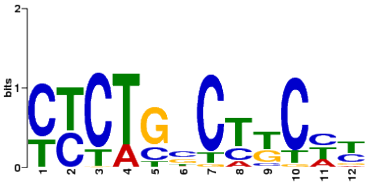	MAF	2.20E-18	190	196	0.97
K4	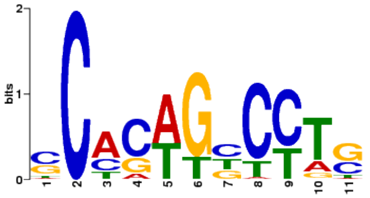	TRIM15	4.10E-10	185	185	1.00
K5 (Motif 1 from Figure 8)	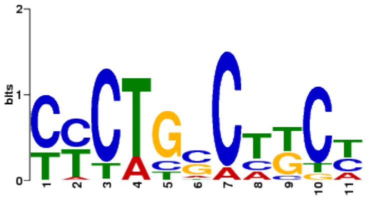	ZNF395	2.30E-17	182	183	0.99
K6	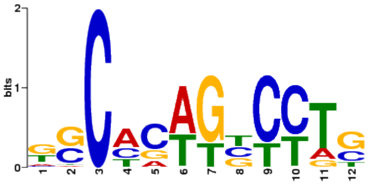	TFEC	1.90E-10	137	137	1.00
K7	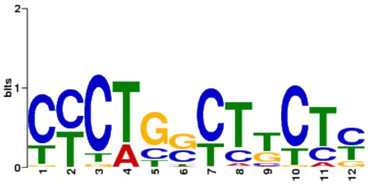	CREB3L3	3.50E-10	125	125	1.00
